# Genetic Variability of Gene Expression in Tomato Fruits Ripened on and off the Vine: Cis-Regulatory Elements Associated with Differential Transcription Patterns in the Most Discrepant Variety

**DOI:** 10.3390/plants15010053

**Published:** 2025-12-24

**Authors:** Javier Hernán Pereira da Costa, Eduardo Daniel Souza Canada, Ana Claudia Ochogavía, Gustavo Rubén Rodríguez, Guillermo Raúl Pratta

**Affiliations:** 1 IICAR-UNR-CONICET, Instituto de Investigaciones en Ciencias Agrarias de Rosario, Campo Experimental Villarino, Universidad Nacional de Rosario, Consejo Nacional de Investigaciones Científicas y Técnicas, Zavalla S2125ZAA, Santa Fe, Argentina; anaochogavia@conicet.gov.ar (A.C.O.); grodrig@unr.edu.ar (G.R.R.); gpratta@unr.edu.ar (G.R.P.); 2 Cátedra de Genética, Campo Experimental Villarino, Facultad de Ciencias Agrarias, Universidad Nacional de Rosario, Zavalla S2125ZAA, Santa Fe, Argentina; 3 Campo Experimental Villarino, Plataforma Agrotecnológica Biomolecular—Facultad de Ciencias Agrarias, Universidad Nacional de Rosario, Zavalla S2125ZAA, Santa Fe, Argentina; eduardo.souzacanada@unr.edu.ar; 4 Cátedra de Química Orgánica, Campo Experimental Villarino, Facultad de Ciencias Agrarias de Rosario, Universidad Nacional de Rosario, Zavalla S2125ZAA, Santa Fe, Argentina

**Keywords:** *S. lycopersicum*, promoter analysis, tomato post-harvest life, tomato fruit quality, stress-related genes

## Abstract

The cultivated tomato (*Solanum lycopersicum* L.) is a self-pollinating species whose fruit ripening is crucial for commercial quality, as many attributes are established during this stage. Fruits ripened on the plant usually have better quality than those ripened off the plant. This study combines traditional molecular techniques (cDNA-AFLP) with bioinformatics tools (PlantCARE and PLACE) to analyze gene expression in fruits ripened on the plant and off the plant from both cultivated and wild genotypes, which differ in shelf life. The goal is to analyze genetic variability in ripening-related transcripts at both ripening conditions and, once identified, the most discrepant variety, to characterize its cis-regulatory elements in the 5′ DNA regions inducing differentially expressed genes. Results revealed wide genetic variability in gene expression according to differentially cDNA-AFLP amplicons detected in both ripening conditions. A strong association among the expressed genes under both ripening conditions and the phenotypic fruit traits related to post-harvest life was found. Though wild genotypes showed the greatest number of amplicons, the most discrepant genotype was the cultivated variety of standard shelf life. The analysis of the promoter in this genotype showed differences in cis-elements between conditions. In shelf-ripened fruits, stress-related elements were predominant and located near the transcription start site, whereas in on-plant ripening fruits, cis-motifs were more abundant farther from the start site. This research provides initial insights into the transcriptional networks regulating ripening and stress responses, offering valuable information for future genetic improvements and post-harvest strategies in tomato cultivation.

## 1. Introduction

The cultivated tomato (*Solanum lycopersicum* L.) is a self-pollinating species in which fruit quality plays a crucial role in production and consumption. To extend the commercialization period, the tomato fruits are harvested at the breaker stage, when partial induction of the genetic network to fully ripen off the vine was achieved. Fruit ripening is a key process for commercialization due to many quality attributes, such as color, aroma, flavor, texture, firmness, and post-harvest life, which are defined during its final stages [1,2]. Fruits ripened on and off the vine differ in some commercially important quality attributes. Hence, delaying the fruit ripening process is a strategy used to increase post-harvest life and extend the marketing time. Several mutants in *S. lycopersicum* are known to affect fruit ripening, extending post-harvest life. Examples include *rin* (ripening inhibitor), *nor* (non-ripening), *Nr* (never ripe), and *alc* (alcobaca) [3]. However, these genes, even in the heterozygous condition, exert negative effects on fruit quality due to their pleiotropic action on metabolic pathways responsible for desirable traits such as flavor, aroma, and texture. In contrast, wild species of the genus *Solanum*, section *Lycopersicon*, often exhibit extensive variability in attributes such as flavor, aroma, coloration, and texture, while also possessing a long post-harvest life [4,5]. Under the hypothesis that in natural conditions, ripening on the vine maintaining organoleptic attributes has been naturally selected to increase the period of effectively attracting frugivorous animals, differential gene expression in fruit ripening on and off the vine must occur among standard and mutant genotypes of the cultivated tomatoes compared to wild tomatoes.

The post-harvest physiological changes in tomato fruits have been extensively studied. Fei et al. [6] showed that detached green tomato fruits could turn red under environmental temperature conditions of about 25 °C, although the fruit quality was affected by lower firmness and content of carotenoids and sugars than that of fruits ripened on the plant. Therefore, given that plant-ripened fruits exhibit better quality than those ripened off the plant, it is of interest to identify the genes that are differentially expressed under these two ripening conditions. Additionally, comparing different genotypes with varying post-harvest shelf lives could enable the identification of genotype-specific genes as well as those common to the ripening process.

Transcriptomic analysis provides a foundation for understanding the regulation of plant growth and development and facilitates the identification of specific control points in metabolism. Complementary DNA amplified fragment length polymorphism (cDNA-AFLP) is a method that has been successfully used to identify genes involved in various processes, such as pathogen resistance in plants and fruit shelf life in tomatoes [7,8]. More recently, some studies [9,10] have employed high-throughput sequencing techniques, such as RNA-seq, to determine differentially expressed genes during fruit ripening. However, the vast amount of data generated complicates both analysis and interpretation, making it challenging to identify specific genes with key roles in ripening from such studies. cDNA-AFLP has traditionally been used to examine gene expression in only one genotype under shifting or contrasting biotic or abiotic stress conditions. Nevertheless, to assess genetic variability for identifying valuable genes in breeding programs, evaluation on more than two genotypes and multiple ripening stages, cDNA-AFLP becomes a more cost-effective alternative to RNA-seq. In fact, we [8] used cDNA-AFLP profiles to make a comparative analysis for the transcriptome at different fruit ripening stages in genotypes that differ in fruit shelf life. While these techniques serve as a preliminary approach, the validation of results invariably relies on the testing of specific genes using additional techniques such as RT-qPCR [11].

The regulation of differential gene expression is a highly complex process that occurs at multiple levels. It involves the coordinated action of cis-regulatory elements present in promoters, which are recognized by trans-acting factors. Today, genomics and transcriptomics have become invaluable tools for conducting studies, identifying and discovering promoters, and predicting their sequences and cis-regulatory elements on a large scale [12]. Bioinformatic tools such as PlantCARE and PLACE enable the in silico analysis of regulatory sequences in promoters in a straightforward and efficient way. The analysis of cis-regulatory elements is important to elucidate the regulatory mechanisms of genes involved in tomato fruit ripening and quality determination. A recent study [13] has reported the key role of cis-elements in the regulation of hormone synthesis, such as ethylene and ascorbic acid, related to tomato fruit ripening and responses to salt stress. For making an effective in silico search, the identification of the most discrepant genotypes respecting the multivariate characterization is necessary. Discrepant genotypes—those with notable differences in genetic or regulatory regions—are valuable sources of novel alleles and regulatory variations that can be harnessed to improve desirable traits. Application of multidimensional statistical techniques, such as Generalized Procrustean Analysis, allows rapid and efficient management of the three-way structured database under study [14]. Particularly in breeding, it is most common to use the concept of “the best ideotype”. The GPA does not quantitatively assess the objects (genotypes in this case), making it difficult to identify one of some of them as “the best”. Hence, the use of “the most discrepant genotype” is a more appropriate concept, given that GPA categorizes objects and indicates discrepancies among variables.

In this context, taking into account that to extend the marketing period, tomato fruits are harvested at the breaker stage, and given that during the final stages of ripening the fruit’s quality traits are defined, it is of interest to study the gene expression profiles during the tomato fruit ripening on and off the vine. Additionally, elucidating the putative mechanisms controlling the expression of these genes in genotypes that differ in quality traits is important. Therefore, the objectives in this study are as follows: (1) to assess differentially expressed genes (DEGs) between on- and off-vine ripening in genotypes with contrasting fruit shelf life; (2) to identify the most discrepant genotypes within the variability for ripening-related phenotypic and molecular attributes; and (3) to characterize the distribution of cis-elements in the promoter of DEGs associated with ripening conditions in the genotypes identified as the most discrepant. 

## 2. Results

### 2.1. Analysis of cDNA-AFLP Expression Profiles of Plant-Ripened and Shelf-Ripened Fruits

In order to assess differentially expressed genes (DEGs) between on- and off-vine ripening in genotypes with contrasting fruit shelf life, cDNA-AFLP profiles were obtained. The analysis of gene expression revealed 3179 transcript-derived fragments (TDFs) across the four evaluated genotypes (CAI: Caimanta cultivar of *Solanum lycopersicum*; LA0722: wild accession of *S. pimpinellifolium*; LA1385: accession of *S. lycopersicum* var. *cerasiforme;* and NOR: accession 804627 (homozygous for the nor mutant gene) of *S. lycopersicum*). On average, 132.4 TDFs were observed per primer combination and genotype (Supplementary Table S1). The highest number of TDFs was detected in the NOR genotype, with a total of 882 TDFs, while the CAI genotype had the lowest number of TDFs, with 694.

**Figure 1:**
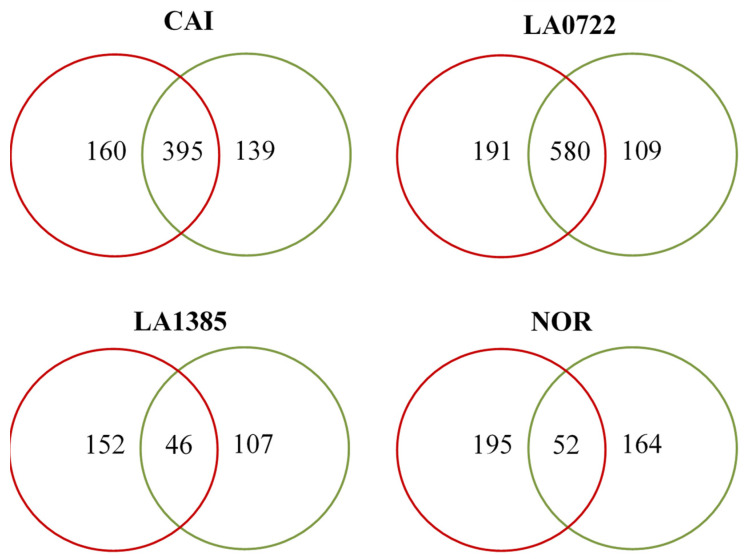
Venn diagrams showing the total and unique number of transcript-derived fragments (TDFs) obtained from AFLP-based transcript profiling using six specific primer combinations in four tomato genotypes. CAI: Caimanta cultivar of *Solanum lycopersicum*; LA0722: wild accession of *S. pimpinellifolium*; LA1385: accession of *S. lycopersicum* var. *Cerasiforme*, NOR: accession 804627 (homozygous for the nor mutant gene) of *S. lycopersicum*. Red circles represent fruit ripened on the plant, and green circles represent fruit ripened on the shelf. Numbers in the overlapping regions indicate monomorphic TDFs (shared between the two ripening conditions), while numbers outside the overlaps represent TDFs exclusive to each ripening condition.

**Figure 2:**
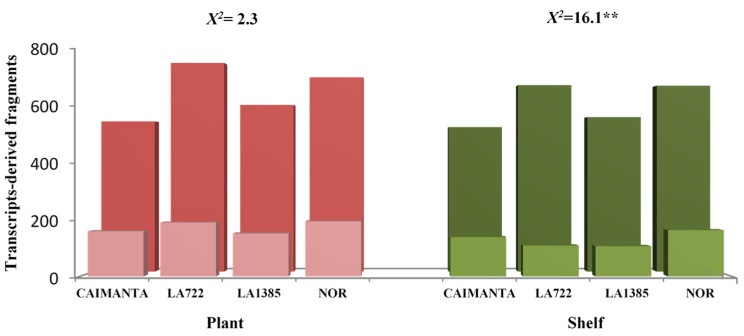
Histogram showing chi-square independence test for total and exclusive transcript-derived fragments obtained by cDNA-AFLP profiles from fruits ripened on the plant and on the shelf. Total and exclusive transcript-derived fragments are represented in the background and in the front, respectively. Caimanta: Caimanta cultivar of *Solanum lycopersicum*, LA0722: wild accession of *S. pimpinellifolium*, LA1385: accession of *S. lycopersicum* var. *cerasiforme*, NOR: accession 804627 (natural mutant in nor gene) of *S. lycopersium*. ** *p* < 0.01.

The comparison of expression profiles between plant-ripened versus shelf-ripened fruits is shown in Figure 1. For all genotypes, the number of monomorphic TDFs was greater than that of polymorphic ones. The total number of TDFs detected in plant-ripened fruits (2660) was higher than that of those ripened on the shelf (2481). Plant-ripened fruits also showed the highest number of exclusive TDFs (698), representing 57.35%, with the NOR genotype having the highest quantity (195) (Figure 1 and Figure 2). LA0722 and LA1385 exhibited the greatest difference in polymorphic genes between those observed in plant-ripened fruits and those found in shelf-ripened fruits (Figure 2). On the other hand, the NOR genotype also had the highest number of exclusive TDFs (164) in shelf-ripened fruits (Figure 1 and Figure 2). 

The chi-square analysis of independence showed that both the total and exclusive TDFs detected in plant-ripened fruits are independent of the genotype. Conversely, both the total number and the number of exclusive TDFs observed in shelf-ripened fruits were genotype dependent (see the chi-square value in Figure 2).

### 2.2. Association Analysis by Generalized Procrustes Among cDNA-AFLP Expression Profiles and Traits Related to Fruit Ripening and Identification of the Most Discrepant Genotype

**Figure 3:**
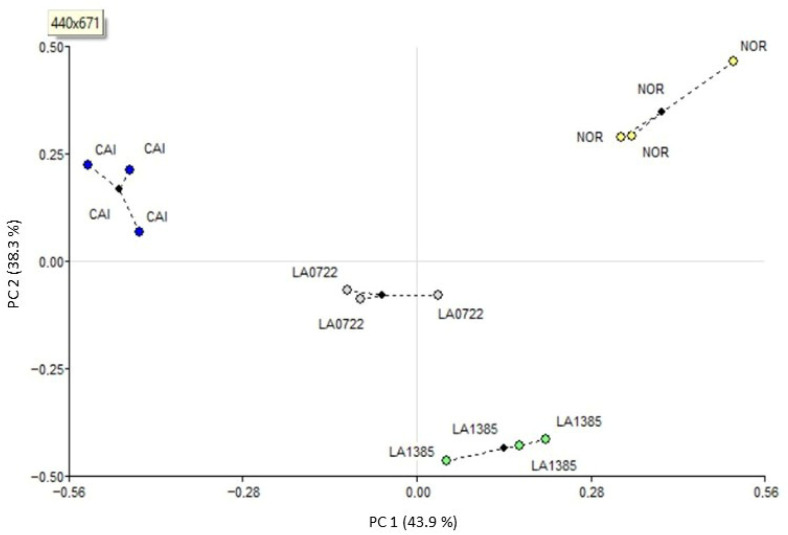
Generalized Procrustes Analysis (GPA) using three groups of variables. Group 1: post-harvest life, days elapsed from the mature green stage to the breaker stage, and days elapsed from the breaker stage to the ripe red stage. Group 2: cDNA-AFLP profile obtained from plant-ripened fruits. Group 3: cDNA-AFLP profile obtained from shelf-ripened fruit. Black points represent consensus configurations, and colored points indicate the original configuration for each group. CAI: Caimanta cultivar of *Solanum lycopersicum*, LA0722: wild accession of *S. pimpinellifolium*, LA1385: accession of *S. lycopersicum* var. *cerasiforme*, NOR: accession 804627 (natural mutant in nor gene) of *S. lycopersium*.

To identify the most discrepant genotypes within the variability for ripening-related phenotypic and molecular attributes, a Generalized Procrusted Analysis (GPA) was carried out. GPA is a graphical method that adjusts the variation in different dimensions of data. In this case, genotypes as objects, phenotypic variation, molecular polymorphism on the vine, and molecular polymorphism off the vine are distinct and independent dimensions of variation. Therefore, GPA not only identifies the most discrepant object (genotype) based on the consensus values—visually represented as the point most distant from the mean values in the biplot—but also highlights the object with the greatest variation across dimensions. This is indicated by the residuals and the ability to visualize the three points representing phenotypic traits, molecular variation on the vine, and molecular variation off the vine. Figure 3 shows the biplots obtained from GPA based on the expression profiles and phenotypic traits. The highest sum of squares of consensus was observed for CAI and NOR genotypes among the three groups of variables (phenotypic expression patterns from plant- and shelf-ripened fruits). This result indicates a strong association among the expressed genes under both ripening conditions and the phenotypic fruit traits evaluated, as well as a distant placement of these genotypes from the coordinate origin in the biplot (Figure 3 and Supplementary Table S2). Also, CAI and NOR had the greatest sum of squares for residual, indicating variability for the three datasets evaluated in each genotype (gene expression on the vine, gene expression of the vine, and phenotypic traits). Together with the three clear points in the biplot for CAI, this result pointed out that this cultivated genotype was the most discrepant within the available genetic variability in the present experiment. CAI was the most discrepant genotype, relevant to selecting it for modifying gene frequencies in a breeding program, as previously mentioned in the Introduction section, but also the genotype evidencing a greater variability for cDNA-AFLP profiles. This fact was important for bioinformatics analyses in order to find different sequences at the genome level. Hence, subsequent sequencing and in silico analysis will be achieved on CAI differentially expressed cDNA-AFLP amplicons.

Additionally, when a GPA was made by individual primer combination, the highest sum of squares for CAI and NOR were observed with combinations A and B (Supplementary Table S2). Therefore, sequencing and identification of polymorphic bands derived from primer combinations A and B at different ripening sites could help identify genes that explain this behavior and potentially relate to the variation in phenotypic traits observed among these genotypes. The lowest sum of squares consensus was observed for group 1 of variables, so the genotypes exhibited greater variability for the phenotypic traits, followed by the expression profile in plant-ripened fruits (Supplementary Table S2). In the case of the residual sum of squares, it was observed that the genotype LA0722 exhibited greater discrepancies between the three groups of variables. 

GPA identifies the most discrepant object (genotype) based on the consensus values—visually represented as the point most distant from the mean values in the biplot. The object with the greatest variation across dimensions is indicated by the residuals and the ability to visualize the three points representing phenotypic traits, molecular variation on the vine, and molecular variation off the vine.

### 2.3. Sequence and In Silico Analysis in CAI and NOR Genotypes

#### Identification and Validation of Differentially Expressed Gene from Transcript-Derived Fragments

In order to validate differential gene expression patterns, a sample of genes was subjected to quantitative PCR analysis. A set of twenty well-defined and distinguishable bands, showing a clear polymorphism pattern between plant- and shelf-ripened fruits, was eluted from the polyacrylamide gel and sequenced (Supplementary Table S3). These polymorphic bands were used as validation of the profiles obtained by cDNA-AFLP. Thirteen bands corresponded to the CAI genotype, three to LA1385 and NOR, and one to LA0722. The two databases used, SGN (www.solgenomics.net) and NCBI (http://www.ncbi.nlm.nih.gov/), enabled the identification of ten genes (50%) with putative functions based on sequence homology. These genes corresponded to bands eluted from only two of the four genotypes analyzed. Nine were from CAI and one from NOR. No genes were identified for the wild genotypes L0A722 and LA1385. Table 1 describes the genes identified by sequencing, indicating the chromosome location, the strand, position, and size in base pairs, as well as their function and the pathway in which they participate (https://www.uniprot.org).

**Figure 4:**
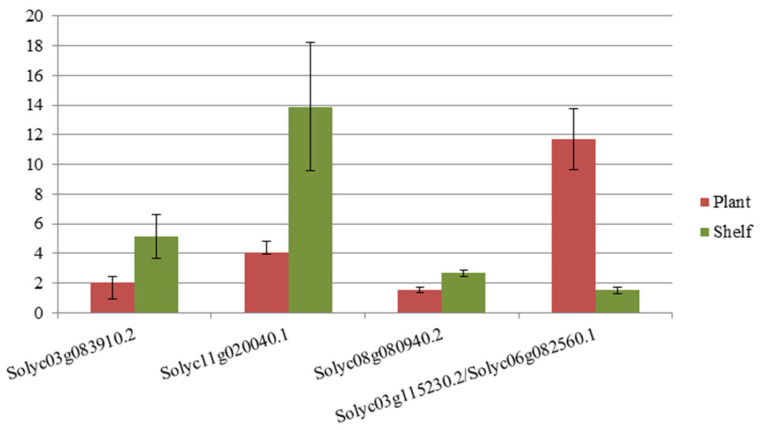
RT-qPCR validation of genes with differential expression between plant-ripened and shelf-ripened fruits. Relative expression means and standard errors are represented. Normalized expression was calculated using the SAND reference gene.

As mentioned above, an RT-qPCR analysis was performed to validate the reliability and the differential gene expression detected by our cDNA-AFLP analysis. Specific primers were developed for the genes described in the above section. Eight out of the ten identified genes were used, including the two paralogous genes (Table 2). Three genes showed significant differential expression by RT-qPCR (Supplementary Table S4). Subsequent analyses were focused on the genes whose differential expression was both significant and consistent with the observations made by cDNA-AFLP (Solyc08g080940.2, Solyc03g115230.2, and Solyc06g082560.1) or those with a *p*-value close to the significance level of 0.05 (Solyc03g083910.2 and Solyc11g020040.1) (Figure 4). Solyc03g083910.2.1, Solyc11g020040.1.1, and Solyc08g080940.2.1 are genes with expression in shelf-ripened fruit, while Solyc03g115230.2.1 and Solyc06g08250.1.1 are expressed genes only in plant-ripened fruits.

### 2.4. Identification of Promoter Regions and Analysis of Cis-Regulatory Elements

To characterize the distribution of cis-elements in the promoter of DEG associated with ripening conditions in the genotypes identified as the most discrepant, the analysis of the promoter region for the validated genes from the previous step was carried out. A variation in the lengths of the 5′UTR regions of these genes was observed. Notably, an intron was found in the 5′UTR of the Solyc03g115230 gene, whereas the 5′UTR could not be identified for the Solyc06g082560 gene (Table 3). PlantCare software (http://bioinformatics.psb.ugent.be accessed on 15 August 2023) was used to predict the presence of known cis-regulatory elements in plant genes. The identified cis-regulatory elements and their predicted functions are listed in Supplementary Table S5. Fifty-four different types of cis-regulatory elements were found. Forty-four were detected in shelf-ripened fruits, while 31 cis-regulatory elements were identified in plant-ripened fruits. Thus, 22 (40.7%) cis-regulatory elements were common to both groups (Supplementary Figure S1). Twenty-two (50%) of the 44 elements found were exclusive to shelf-ripened fruits, that is, they were not found in genes expressed in plant-ripened fruits, while 9 (29%) of the 31 elements found were exclusive to plant-ripened fruits.

**Figure 5:**
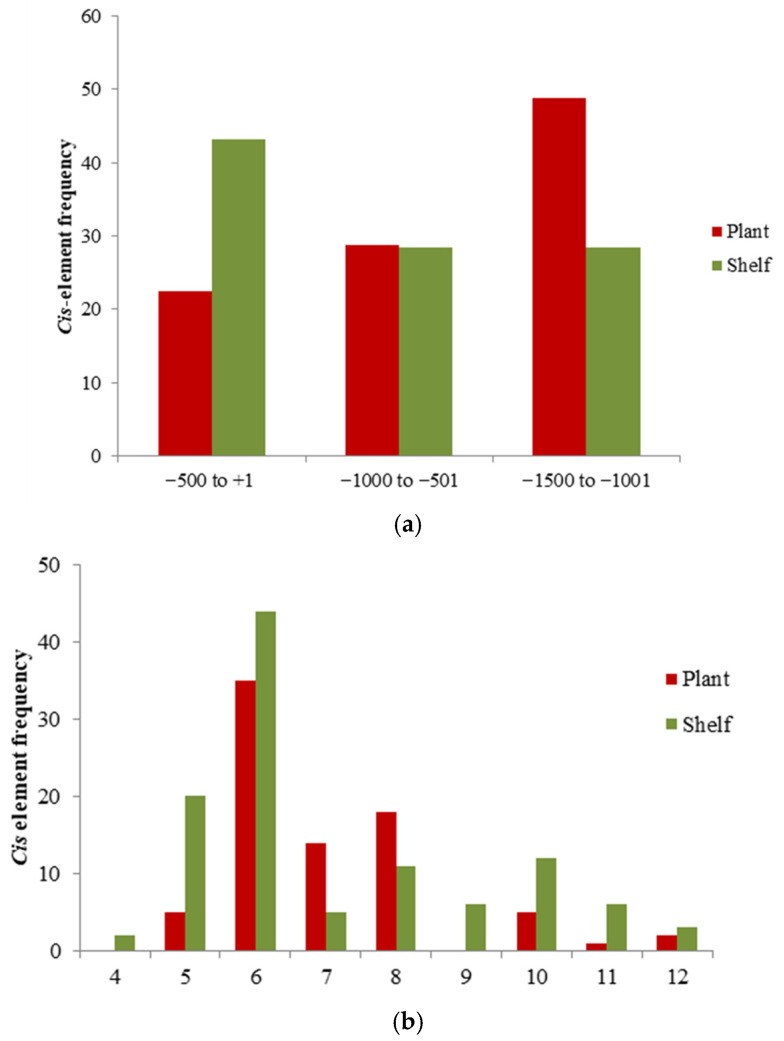
(**a**) Distribution of cis-regulatory elements every 500 bp in the promoter region from the ATG codon of polymorphic genes detected in shelf-ripening and plant-ripening fruits for all genotypes together (Caimanta cultivar and NOR mutant of *Solanum lycpersicum*). (**b**) Histogram showing the frequencies of cis-element sequences of different lengths (from 4 to 12) in polymorphic genes between fruit ripened on the shelf and fruit ripened on the plant.

By dividing the 1500 bp promoter region into three sub-regions of 500 bp upstream from the start codon, it was found that in shelf-ripened fruit, a higher percentage (42%) of cis-motifs were located near the translation start site, between −501 and +1 bp (Figure 5a). In contrast, in plant-ripened fruits, 48.8% of the cis-elements were located further away from the ATG codon (between −1500 and −1001). Regarding motif length, it was found that motifs of 6 bp in length were the most abundant, with 40.4% in shelf-ripened fruits and 43.8% in plant-ripened fruits (Figure 5b).

**Figure 6:**
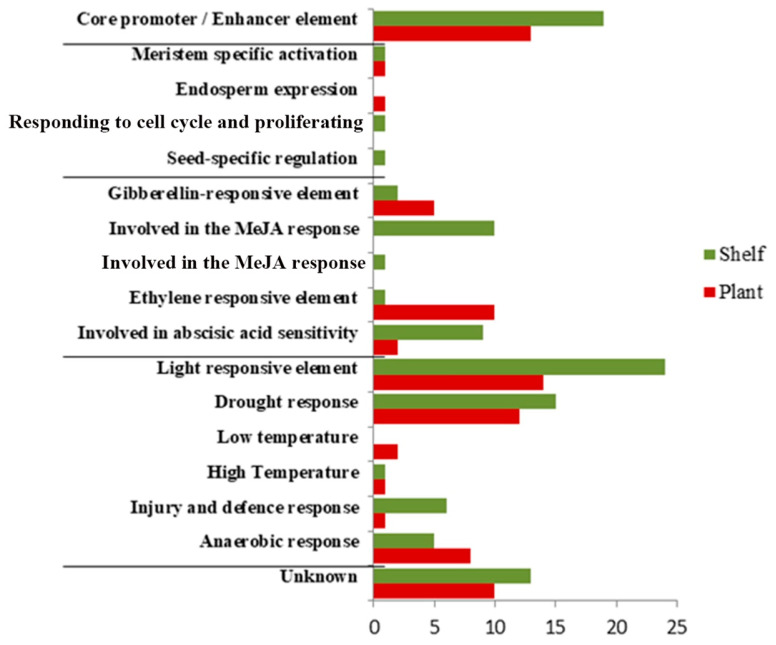
Frequency of the motifs identified in the genes expressed in shelf-ripened and plant-ripened fruits grouped by biological function. Axis y represents the biological functions, and axis x indicates the number of motifs. Unknown motifs: AAGAA-motif, AP-1, Myc, Unnamed_1, and Unnamed_6.

Concerning the function, the largest percentage of cis-elements (~48%) belonging to the functional category of stress response was found in both ripening conditions (Figure 6 and Supplementary Table S6). The second most represented functional category, accounting for 21% of the identified cis-regulatory elements, consisted of hormone-related regulatory motifs (Figure 6). In shelf-ripened fruits, cis-regulatory elements related to methyl jasmonate response (9%) and ABA-responsive elements (8%) were more prevalent. Methyl jasmonate response elements (CGTCA-motif and TGACG-motif) were found in two (Solyc11g020040 and Solyc08g080940) of the three analyzed genes, while ABA-responsive elements were found in one gene expressed in shelf-ripened fruits (Solyc08g080940), represented by ABRE, ABRE3a, and ABRE4, and in one gene expressed in plant-ripened fruits (Solyc03g115230, ABRE cis-element).

## 3. Discussion

### 3.1. Analysis of cDNA-AFLP Expression Profiles of Plant-Ripened and Shelf-Ripened Fruits

The number of TDFs revealed by cDNA-AFLP was consistent with in silico simulations estimating TDFs based on primer combinations, as reported by [15]. Additionally, these results prove that the used restriction enzymes (ApoI and MseI) were effective in generating the expression profile of each genotype at each fruit ripening condition. Hence, we can suppose that each TDF matches only one transcript derived from one gene. The differences observed between NOR and CAI regarding the total amount of TDFs (Supplementary Table S1) can be explained by considering that, by the end of the seven-day analysis, CAI would be in the fruit softening phase, fully red and ripe. This stage is recognized as the conclusion of the ripening cycle, which is estimated to occur between 5.26 and 7.96 days, according to [8]. Furthermore, in this stage, there is a decrease in metabolic processes and, therefore, a decrease at the transcriptomic level. On the other hand, in the NOR genotype and the wild LA0722 and LA1385 genotypes, carrying genes that extend the fruit shelf life [16,17], the fruits would still be metabolically more active at the time of RNA extraction at seven days after collection of pericarp, explaining their higher total number of TDFs (expressed genes).

Higher TDF numbers detected in shelf-ripened vs. plant-ripened fruits were consistent across all genotypes, with a greater number of total TDFs recorded in plant-ripened fruits (Figure 1). It has been observed that ethylene concentration is unusually high in fruits attached to the plant (5.4 ppm on average, ranging from 2 to 13 ppm depending on cultivars and seasonal conditions). In contrast, when tomatoes detached from the plant, ethylene levels dropped to a lower level (1.4 ppm on average) [18,19]. Therefore, the reduction in total FDTs in shelf-ripened fruits (Figure 1) could be attributed to the decrease in this plant hormone.

Wild accession and even the NOR mutant had the highest number of exclusive TDFs (Figure 1 and Figure 2). This result, a priori, would not be expected, as the NOR mutant blocks the normal ripening process. Since NOR encodes a transcription factor, it is expected to halt the expression of genes in early stages during fruit ripening [20]. This higher number of polymorphic TDFs would indicate that in the NOR genotype, other active genes are involved that escape the modulation of the transcription factor encoded by the nor gene. In fact, Gao et al. [21] observed that overexpression of the NAC-NOR gene in the nor mutant did not restore the full ripening phenotype. After all, complex and crucial biological processes, such as ripening, beyond linear and two-dimensional models, are often regulated by a highly redundant transcriptional network [22].

### 3.2. Associations Among Molecular and Phenotypic Variability

The result of the independence test (Figure 2) suggests a possible association between differentially expressed genes and the contrasting shelf life values among the four genotypes. At the same time, the high heritability observed by Pereira da Costa et al. [8] for post-harvest life and days elapsed from breaker to red ripe stage (0.65 and 0.50, respectively) indicates a strong genetic determination of these traits. 

The association between the profiles of expressed genes in plant- and shelf-ripened fruits is further strengthened by the GPA results (Figure 3 and Supplementary Table S2), based on the high proportions of consensus detected among phenotypic traits and expression profiles, particularly for the CAI and NOR genotypes. Additionally, the A primer combination yielded the highest consensus, which explains and justifies the greater number of sequenced bands for this combination. Del Medico et al. [23] established the utility of GPA as a three-way data analysis to capture the genetic variability and proposed this analysis as an adequate tool to assist breeding programs. In agreement, other studies had reported the possibility of detecting QTLs from the high consensus found by GPA between phenotypic fruit traits and molecular data of ADN [24,25]. Our results add new evidence regarding the efficiency of GPA in establishing the association between phenotypic data and gene expression data. In particular, it has enabled us to make the in silico search for cis-regulatory elements more efficient by identifying the most discrepant genotypes.

### 3.3. Identification and Validation of Differentially Expressed Gene-From Transcript Derived Fragments

A clear skew toward the cultivated genotypes (CAI and NOR) was found when putative functions based on sequence homology were searched. This result is likely due to the preferential use of *S. lycopersicum* as a model for ripening studies in the past [26]. The FDTs with known functions were categorized according to their roles. Out of the genes identified in the CAI genotype, three genes related to stress and one to cell structure were found in plant-ripened fruits. While for shelf-ripened fruits, the gene functions were associated with metabolism, one to transporters, one to cell structure, and two to stress response. CAI.EST.A.6 and CAI.PL.B.2 are two polymorphic genes belonging to the CAI genotype, the first found in shelf-ripened fruits and the second when it ripened on the plant, both identified as the same gene, Solyc11g020040.1 (Table 1). Additionally, the sequenced fragment CAI.PL.E.1 allowed the identification of two genes (Solyc03g115230 and Solyc06g082560), which are paralogs (Gramene, Plaza 4.5). Both genes share the same function as ClpB Chaperone Protein/ATPase. The only gene identified on plant-ripened fruits for the NOR genotype (NOR.PL.A.1) encodes a ribosomal protein.

The validation of differential gene expression made by RT-qPCR was in agreement with the profiles obtained by cDNA-AFLP, except for Solyc03g112910.2, which showed a higher expression level in shelf-ripened fruits than in plant-ripened fruits, which was contrary to the results observed by cDNA-AFLP. The inconsistency in expression levels between RT-qPCR and cDNA-AFLP for some genes may be due to several factors, one of which could be the presence of paralogous genes in the genome [7], or could indicate a complex regulated gene family, which can only be identified using cDNA-AFLP [27].

### 3.4. Identification of Promoter Regions and Analysis of Cis-Regulatory Elements

A variation in the length of 5’UTR regions of the genes, as well as the amount and type of cis-regulatory elements, was found. It is well known that each gene possesses a unique combination of cis-acting elements within its 5′ regulatory sequence, which determines its temporal and spatial expression and contributes to the regulation of numerous biological processes and stress responses [28]. 

The closest sequence upstream of the gene or proximal promoter contains the major regulatory elements that can influence the level and/or pattern of expression of the gene [29]. The distal upstream sequence or distal promoter may contain other regulatory elements that exert a weaker control on transcriptional gene regulation [30]. By dividing the 1500 bp promoter region into three sub-regions of 500 bp upstream from the start codon, it was found that in unripened fruit, a higher percentage (42%) of cis-motifs were located near the translation start site, between −501 and +1 bp (translation start site), suggesting that the cis-regulatory elements of these genes may be regulated in the proximal region (Figure 5a). In contrast, in plant-ripened fruits, 48.8% of the cis-elements were located further away from the ATG codon, suggesting that regulation takes place in the distal region. Similar results, where motifs within the promoter region are primarily located in one region or the other, were reported by [28] in an in silico analysis of cis-regulatory elements of sucrose transporter genes in rice (Oryza sativa Japonica) and Arabidopsis thaliana. Regarding motif length, the motifs of 6 bp in length were the most abundant, with 40.4% in shelf-ripened fruits and 43.8% in plant-ripened fruits (Figure 5b). This finding is consistent with the same analyses of genes in other species such as *A. thaliana* and rice [31].

Accordingly, the TATA-box elements in genes expressed in shelf-ripened fruits were positioned closest to the TSS, with distances of 25 bp (Solyc03g083910), 84 bp (Solyc11g020040), and 107 bp (Solyc08g080940). In genes expressed in plant-ripened fruits, no proximal TATA-box was found for Solyc03g115230 (TATA-less). The absence of a TATA-box is possible, as various genomic studies have found that many groups of genes (e.g., housekeeping genes) lack the TATA-box [32,33,34]. For Solyc06g082560, where the 5′ UTR region could not be predicted, a TATA-box was identified at a distance of 251 bp from the translation start site.

The classification of these cis-regulatory elements into functional categories (Supplementary Table S6) allowed us to analyze which regulatory motifs are contained in the genes identified at both ripening conditions. The largest percentage of cis-elements (~48%) belonging to the functional category of stress response was found in both ripening conditions (Figure 6), indicating that the expression of the polymorphic genes analyzed here could be related to and regulated by stress conditions. It is well known that fruit ripening is itself an oxidative process that also involves water loss and softening. In fact, four of the five genes identified and validated in this study are associated with stress situations (Table 1). Solyc11g020040 is a DNA k chaperone that belongs to the heat shock protein 70 family, which is stress-inducible and whose primary function is to prevent nonspecific protein aggregation during the stress response [35]. These proteins are induced not only by heat stress but also during development [36,37] and fruit ripening [38]. Solyc08g080940 corresponds to a glutathione peroxidase enzyme (GPXle-1), which is located in the cytoplasm and responds to oxidative stress by protecting cells and enzymes from oxidative damage by catalyzing the reduction of hydrogen peroxide, lipid peroxides, and organic hydroperoxides by glutathione (www.uniprot.com). On the other hand, Solyc03g115230 is a heat shock protein, and Solyc06g082560 is a ClpB chaperone protein; both are also related to stress and likely play an important role in thermotolerance.

As previously discussed, three of the differentially expressed genes—one expressed in shelf-ripened fruits and the other two in plant-ripened fruits (Solyc03g115230.2 and Solyc06g082560.1)—encode heat shock proteins (HSPs). These proteins are associated with high-temperature stress, which helps protect and refold cellular proteins and maintain cellular homeostasis for plant survival [39]. Generally, genes involved in these mechanisms contain motifs known as heat shock elements (HSEs) that bind to heat shock transcription factors (HSFs) and are upregulated during heat stress. HSEs have been found to be consistently conserved in the regulatory regions of many heat-induced genes [40]. Both Solyc11g020040 (from shelf-ripened fruits) and Solyc03g115230 (from plant-ripened fruits) encodes heat shock proteins and are related to high-temperature stress. Each one contains only one heat-related motif, the stress response element (STRE), which specifically binds to nuclear proteins activated by heat shock [41]. Curiously, no heat-related motifs were identified in Solyc06g082560, despite its known association with thermotolerance. Concurrently, in a study conducted with a tomato genotype (E42) tolerant to heat stress [42], 35 genes associated with this phenotype were identified, and no HSE motifs were found in the promoter region analysis of those genes. A separate study carried out on tomatoes by [43] reported similar results. They analyzed the presence of two HSEs associated with small heat shock protein (sHSP) families and found that a gene highly regulated during fruit ripening lacked the analyzed HSEs in its promoter region. Another motif, such as CTAGA, has been proposed as a potential binding site for transcription factors that enable the gene response during fruit ripening. This suggests the existence of alternative regulatory systems for the stress response. Thus, the absence or scarcity of HSEs of these three heat stress-associated genes in this study may indicate regulation through an alternative, unidentified system.

The second most represented functional category among cis-elements was hormone-related regulatory motifs. Numerous studies have shown that auxin, ABA, and jasmonic acid (JA) also affect the expression of genes involved in ethylene biosynthesis and other aspects of the ripening control network [44]. Among the cis-regulatory elements in this category, three of the most represented are related to plant hormones, which are key regulators of stress response pathways [40], including ethylene, JA (jasmonic acid), and ABA (abscisic acid). Reference [9] found that endogenous ABA delays over-ripening in detached fruit. Since not all gene expression patterns are regulated by a single cis-regulatory element but rather by a combination of different regulatory elements that exert different effects at different times or in different cells/tissues [45], the cis-regulatory elements in these two categories could be related and potentially cooperate.

## 4. Materials and Methods

### 4.1. Evaluation of Gene Expression by cDNA-AFLP

#### 4.1.1. Plant Material

Four different tomato genotypes exhibiting varying fruit shelf life are assessed in this study. The ‘Caimanta’ variety (Argentinean elite cultivar, hereafter called CAI) and the accession 804627 (homozygous for the NOR mutant gene, hereafter referred to as NOR), both of *S. lycopersicum*, were included, representing normal and delayed fruit ripening, respectively. Additionally, two wild relatives—LA1385 of *S. lycopersicum* var. *cerasiforme* and LA0722 of *S. pimpinellifolium*—possessing alleles associated with extended fruit longevity [46]—were part of the experiment. The wild and mutant genotypes were provided by the Tomato Genetic Resources Center at the University of California, Davis, CA, USA. These genotypes represent genetic resources contributing to variability for important breeding traits, such as shelf life, fruit quality, and fruit size. The Supplementary Figure S2 displays transverse sections and whole fruits of the four evaluated genotypes. The trial design, management, and cultivation conditions followed the methodology proposed by [8]. Briefly, seeds from these genotypes were germinated in seedling trays and transplanted to the greenhouse after 45 days in a completely randomized design. Ten plants by genotype were distributed with a distance between plants of 40 cm and row spacing of 1 m. The genotypes were evaluated in a greenhouse. Throughout the cultivation period, climatic factors such as temperature, humidity, and irrigation were consistent for all genotypes, minimizing their potential influence on the experimental outcomes. Field assays were conducted at the Experimental Station ‘José F. Villarino’ (33° S and 61° W, Universidad Nacional de Rosario, Argentina). Tissue sampling and total RNA isolation.

One fruit from three different plants (biological repetitions) per genotype was labeled on the plant at the breaker stage. Three additional fruits from the same plants were harvested and stored on a shelf at room temperature (25 ± 3 °C) with an average moisture of 60 %. In total, six fruits per genotype were obtained. After seven days, the pericarps from all six fruits were separated and immediately flash-frozen in liquid nitrogen. Total RNA was extracted from 500 mg of pericarp using TriPure Isolation Reagent, following the manufacturer’s instructions (Roche, Basel, Switzerland). A mortar and pestle were used to powder 500 mg of pericarp with 1 mL of TriPure Isolation Reagent. Then, an equal volume of chloroform was added to separate the aqueous phase, containing RNA. Finally, RNA was precipitated with 0.5 mL of isopropanol.

#### 4.1.2. cDNA Synthesis and Obtaining of cDNA-AFLP Profiles

The synthesis of the first strand of complementary DNA (cDNA) was performed using 1 μg of total RNA, following the protocol provided by ImPromII™ Reverse Transcriptase (Promega, Madison, WI, USA). The processes for first and second strand synthesis, adapter sequence addition, ligation, and amplification were conducted according to [8] and [47]. Primers +0 and primer +1 were employed for pre-amplification and selective amplification, respectively (primer sequences are provided in Supplementary Material, Table S7). The primer combinations designated for selective amplification included the following: A (Apo11-Mse37), B (Apo11-Mse38), C (Apo12-Mse37), D (Apo12-Mse38), E (Apo13-Mse37), and F (Apo13-Mse38). The restriction enzymes ApoI and MseI were chosen based on the study by [14]. The resulting amplified fragments were separated using 5% denaturing polyacrylamide gels at room temperature and visualized with a commercial silver staining kit (Silver Sequence™ Staining Reagents, Promega, Madison, WI, USA). Gel electrophoresis was conducted at a constant power of 50 W for 3 h. As an example, Supplementary Figure S3 shows a section of polyacrylamide gel obtained from primer combination A. For each genotype, the presence or absence of bands—referred to as Transcript Derived Fragments (TDFs)—was recorded. A TDF was considered present if it appeared in at least two biological replicates; absence was determined similarly. A first comparison was made between ripening sites for each genotype, followed by a comparison among genotypes for each ripening site.

### 4.2. Association of cDNA-AFLP Amplicons at Both Ripening Conditions and Quantitative Traits and Identification of the Most Discrepant Genotypes

An integrated Generalized Procrustes Analysis (GPA) was performed to evaluate the association between the phenotypic data described below and the expression profiles obtained for each primer combination separately. For this, matrices of 1 s and 0 s were constructed based on the band profiles obtained in the four genotypes, for each primer combination separately and for each ripening site (plant- and shelf-ripened fruits), along with a third data matrix compiled from the phenotypic data. The mean values for traits related to fruit ripening, such as post-harvest life, days elapsed from the mature green stage to the breaker stage, and days elapsed from the breaker stage to the ripe red stage, were obtained from [8], who evaluated the same genotypes as in the present study. GPA evaluates both the consensus and the residual values for each genotype, which are also allocated in a biplot for assessing their performance according to the evaluated data [14]. The association is estimated by residual, and the most discrepant genotypes were identified by means of a high consensus (allocation far from the coordinate origin of the biplot) and a high residual (great difference with respect to the evaluated data). In this case, to reduce the dimensionality of the molecular data, a matrix was constructed with the first two principal coordinates obtained from a principal coordinate analysis (PCoA) performed for each of the six primer combinations and each ripening site (plant- and shelf-ripened fruits). Thus, the data matrix was composed of 24 principal coordinates, 12 derived from the band profile of fruits ripened on the plant and 12 from fruits ripened on the shelf, in addition to the three phenotypic variables (see Supplementary Table S2).

Also, once the most discrepant genotypes were identified, various GPAs for individual primer combinations were performed to evaluate according to the corresponding consensus and the residual. In this step, the objective was the selection of the primer combination evidencing a great amount of polymorphism level to elute amplicons for sequencing and in silico analysis.

### 4.3. Analysis of Is-Regulatory Elements in the Most Discrepant Genotypes

#### 4.3.1. Validation of cDNA-AFLP Amplicons by Reverse Transcription Quantitative PCR

To confirm the differential gene expression observed with cDNA-AFLP, a quantitative reverse transcription quantitative PCR (RT-qPCR) test was performed. The single-stranded cDNA from each sample was diluted to a concentration of 5 ηg/μL and used as the template for RT-qPCR analysis. Primer sequences were designed using Primer3 (http://frodo.wi.mit.edu/primer3/, accessed on 27 March 2023) based on the specific gene sequences identified through BLAST 2.15.0 searches. The PCR reactions were prepared in a total volume of 25 μL, including 12.5 μL of 2X SYBR Green PCR Master Mix (Applied Biosystems; Carlsbad, CA, USA http://www.bio-rad.com/), 10 μM of each primer, 2 μL of the diluted cDNA, and nuclease-free water. Reactions were run on a CFX96 Real-Time Detection System (Bio-Rad, Carlsbad, CA, USA). The thermal cycling protocol consisted of an initial denaturation step at 95 °C for 2 min, followed by 45 cycles of denaturation at 95 °C for 10 s, annealing at 55 °C for 20 s, and extension at 72 °C for 20 s. A final extension step was performed at 72 °C for 5 min. For each biological sample, three technical replicates were analyzed. Relative gene expression levels were calculated using the ΔΔCt method [48], using the SAND gene as the internal control [49]. Primer sequences for target and reference genes are provided in Table 2. SAND is a housekeeping gene, which showed an efficiency of 0.944 from three technical replica assays and high expression stability in various developmental stages, applying three different statistical approaches to expression data [49]. Statistical significance of differences in gene expression was assessed using Student’s *t*-test.

#### 4.3.2. Promoter Sequence Analysis of Differentially Expressed Genes

The Sol Genomics Network (SGN) database was used to extract a genomic sequence of 1500 bp for each gene corresponding to the promoter region, upstream from the translation start site (ATG). The NCBI (National Center for Biotechnology Information internet database site (https://www.ncbi.nlm.nih.gov/), Phytozome (http://www.phytozome.net/), Gramene (http://www.gramene.org), and PLAZA 4.5 dicot platform (http://bioinformatics.psb.ugent.be/plaza/) databases were used to identify the 5′UTR region of each gene. Multiple alignments were performed with CLUSTAL W in BioEdit [50] to compare the 1500 bp promoter sequences with those obtained from these databases. Putative cis-regulatory elements were predicted by scanning the promoter sequences using the online software PlantCARE (http://bioinformatics.psb.ugent.be/webtools/plantcare/html/, accessed on 27 March 2023) [51]. Cis-elements were predicted using default parameters, which require 100% sequence identity to the consensus motif. The parameters used for PlantCARE included the default settings for motif search, with a minimum motif length of 6 base pairs and a maximum of 50 base pairs. The analysis was conducted using the plant-specific database to identify cis-acting regulatory elements. Overlapping or redundant motifs identified within the same region were automatically filtered by the software, and only the first non-overlapping occurrence of each motif was retained for further analysis. A list of all motifs in the promoter sequences was obtained, indicating the sequence, organism of origin (species from which the motifs come), associated function, position, and strand.

## 5. Conclusions

This study represents an initial step toward elucidating the regulatory transcriptional networks that govern ripening and responses to environmental stress, offering valuable insights for future genetic enhancement and post-harvest handling of tomatoes.

Differences in gene expression are associated with ripening conditions—on the plant or on the shelf. In the last condition, both the total number and the subset of exclusively expressed genes detected by cDNA-AFLP are genotype dependent. The expression profile of the NOR genotype suggests the presence of other active genes that may not be modulated by the NOR transcription factor. On the other hand, the high total number of TDFs (expressed genes) detected in wild genotypes indicated that the fruits remain metabolically active for a long period, which is consistent with their extended fruit shelf life.

The cis-regulatory elements of the genes differed between ripening conditions, with expression apparently linked to and regulated by stress, particularly in shelf-ripened fruits. The positioning of most cis-regulatory elements within the promoter region varied for genes differentially expressed under both ripening conditions, potentially explaining the observed gene expression pattern. Those expressed in shelf-ripened fruits have a higher abundance of the cis motif in the proximal region of the promoter. Conversely, genes expressed during fruit ripening on plants have a higher abundance of cis motifs further from start sites. Although further studies are required to validate these results, this work provides a valuable insight regarding the regulatory transcriptional interactions of these genes during development or under environmental stress conditions.

The evaluation of genetic resources, including related wild species and mutant genotypes within cultivated tomato, through gene expression profiles, has demonstrated the existing variability in fruit ripening both within and outside the plant. This variability present in these genetic resources is interesting for breeding programs and makes them potential starting materials for such programs. 

## Figures and Tables

**Table 1:** List and description of differentially expressed genes identified by sequence.

	Identifier/Gene	Cr.	C.	Location Gene/Length (bp)	Function	
1	Solyc03g083910.2	3	-	53851092..5855368/4277	β-fructofuranosidase acid. Involved in the sucrose metabolism pathway, which is part of glycan biosynthesis.	M
2	Solyc12g044820.1	12	+	37714905-37720440/5536	ABC transporter, member of the C 8 family, ATPase-coupled transmembrane transporter activity.	T
3	Solyc11g020040.1	11	+	10015582..10019521/3940	Heat shock protein 70 (HSP70). Unfolded protein binding.	S
4	Solyc08g080940.2	8	+	64084698..64087731/3034	Glutathione peroxidase 1 (GPx). Protects cells and enzymes from oxidative damage by catalyzing the reduction of hydrogen peroxide, lipid peroxides, and organic hydroperoxide by glutathione.	S
5	Solyc06g076940.2	6	+	47818952..47822936/3985	NudC-domain proteins. Unfolded protein binding.	CS
6	Solyc11g020040.2	11	+	10015582..10019521/3940	Heat shock protein (HSP70). Unfolded protein binding.	S
7	Solyc03g115230.2	3	-	65011966..65016121/4156	ClpB/ATPsa chaperone protein. ATP binding. ATPase associated with various cellular activities.	S
8	Solyc06g082560.1	6	+	48340001..48342565/2565	ClpB/ATPsa chaperone protein. ATP binding. ATPase associated with various cellular activities.	S
9	Solyc03g112910.2	3	+	63189344..63204073/14730	Pantothenate kinase. Catalyzes the phosphorylation of pantothenate, the first step in CoA biosynthesis. It may play a role in the physiological regulation of intracellular CoA concentration.	CS
10	Solyc06g064630.2	6	-	40270353..40272844/2492	Ribosomal protein L15	CS

1-CAI.EST.A.2, 2-CAI.EST.A.5, 3-CAI.EST.A.6, 4-CAI.EST.A.7, 5-CAI.EST.B.1, 6-CAI.PL.B.2, 7-CAI.PL.E.1, 8-CAI.PL.E.1, 9-CAI.PL.F.1, and 10-NOR.PL.A.1. CAI: Caimanta cultivar of *Solanum lycopersicum*. NOR: mutant nor (804627) of *S. lycopersicum*; EST: shelf-ripened fruit; PL: plant-ripened fruit; Cr: Chromosome number; C: Strand; M: metabolism-related function; T: transport-related function; S: stress-related function; CS: cellular structure-related function.

**Table 2:** Specific primers designed with Primer3 using the sequence of the genes identified by sequence comparison with databases. In silico PCR results verify the specificity of the primers.

Identifier/Gene	Forward Primer	Reverse Primer	PCR In Silico
Solyc03g083910.2	TACCTGTGTTGGACGGTGAA	TCGTGCTGCTCCATTTACTG	Solyc03g083910.2
Solyc12g044820.1	TTTCGCCTGGTAGAGCCTTA	GTTCGAACACTCCCCTTGAA	Solyc12g044820.1
Solyc11g020040.1	TACAAGGGCCAAGTTTGAGG	GAACAGCCGGTATTCGTGTT	Solyc11g020040.1
Solyc08g080940.2	ACCAGTTTGGTGGACAGGAG	AAGAACCCACCTTTGCTTGA	Solyc08g080940.2
Solyc06g076940.2	CCCAGTGAAGACCGATTGTT	GGAACCTTTGAAGCATGAGC	Solyc06g076940.2
Solyc03g115230.2/Solyc06g082560.1	ATACGGTGCCATCCAAGAAG	CATTCTGGCCAAGCCTAGAG	Solyc03g115230.2
	CTAGGCTTGGGCAGAATGAG	AGTTGGTTGTTGTGGCCTTC	Solyc06g082560.1
Solyc03g112910.2	TAGGAGCAAGGTAGGCAGGA	GAATTGAACTCCCAGGCAAA	Solyc03g112910.2
Solyc06g064630.2	GAGGAAGAAGCAGTCGGATG	TGCCTTGTCAGGACGTGTAG	Solyc06g064630.2

**Table 3:** Characterization of the differentially expressed genes validated by RT-qPCR, identified in the Caimanta cultivar of *Solanum lycopersicum*.

Treatment	Transcript ID/Length (bp)	5′UTR Before ATG ^a^	N° of Exons	Region CDS ^b^	CDS Length (bp)	Protein Size (aa)
Shelf	Solyc03g083910.2.1/2299	1–82	7	83–4011	1947	649
	Solyc11g020040.1.1/2079	1–317	8	318–4258	2079	693
	Solyc08g080940.2.1/1022	1–36	6	37–1059	720	240
Plant	Solyc03g115230.2.1/3194	1–24/527–633 ^c^	7	701–3831	2736	912
	Solyc06g082560.1.1/2565	-	1	-	2565	855

^a^ This indicates the position of the transcription start site (TSS). ^b^ This indicates the position of the translation start site (ATG). ^c^ Intron in the 5′UTR area. ID: Identifier. CDS: coding region of a gene. bp: base pair. aa: number of amino acids.

## Data Availability

Data supporting reported results can be found at https://ri.conicet.gov.ar/ (accessed on 27 March 2023).

## Supplementary Materials

The following supporting information can be downloaded at: https://www.mdpi.com/article/10.3390/plants15010053/s1. Table S1: Amount of transcript-derived fragments detected by cDNA-AFLP using six specific primer combinations of fruits of four tomato genotypes from plant-ripened and shelf-ripened fruits; Table S2: Analysis of variance from Generalized Procrustes Analysis (GPA) using three groups of variables. Group 1: phenotypic traits such as post-harvest life, days elapsed from the mature green stage to the pink stage, and days elapsed from the pink stage to the ripe red stage. Group 2: first and second principal coordinates building from cDNA-AFLP profiles derived from each primer combination (A, B, C, D, E, F) in plant-ripened fruits. Group 3: first and second principal coordinates building from cDNA-AFLP profiles derived from each primer combinations (A, B, C, D, E, F) in shelf-ripened fruit., Table S3: Sequences of differentially expressed transcript-derived fragments (TDFs), eluted from polyacrylamide gel and obtained through cDNA-AFLP profiling with six primer-specific combinations; Table S4: Relative gene expression tested by RT-qPCR for each gene identified by sequencing of bands eluted from cDNA-AFLP profiles; Table S5: Sequence and function of cis-regulatory elements identified in the 1.5 kb upstream promoter region from five tomato (*Solanum lycopersicum*) genes that were differentially expressed in shelf-ripened fruit (1- Solyc03g083910.2, 2- Solyc11g020040.1, 3- Solyc08g080940.2) and in plant ripened fruit (4- Solyc03g115230.2 and 5- Solyc06g082560.1); Table S6: Functional categories to which the cis-regulatory elements found in genes with differential expression in tomato fruits ripened on the plant and shelf; Table S7: Restriction enzymes, adapters, pre-amplification and specific primer combinations used for cDNA-AFLP profiling of RNA; Figure S1: Amount of cis-regulatory elements into promoter regions of differentially expressed in fruit ripening on plant (red circle) and shelf (green circle). Figure S2: transverse sections and whole fruits of the four evaluated genotypes. Figure S3: Section of a 5 % denaturing polyacrilamide gel visualized with a commercial silver staining kit. The gel shows the cDNA profile obtained for the cultivar Caimanta of *Solanum lycopersicum* from primer combination A (Apo11-Mse37).
