# Supplementary material for: Genetic Variability of Gene Expression in Tomato Fruits Ripened on and off the Vine: Cis-Regulatory Elements Associated with Differential Transcription Patterns in the Most Discrepant Variety
Source: Plants (Basel). 2025 Dec 24;15(1):53. doi: 10.3390/plants15010053 (PMC12787370; doi:10.3390/plants15010053)
Supplement: Supplementary file 1 [file plants-15-00053-s001.zip › Figure S1.pdf]

Genetic variability for gene expression in tomato fruits ripened on and off the vine: cis-regulatory elements are associated with differential transcription patterns in the most discrepant variety

Javier Pereira da Costa[1,2,*]; Eduardo Souza Canada[3]; Ana Ochogavía[1,4]; Gustavo Rodríguez[1,2]; Guillermo Pratta[1,2]

[1]IICAR-UNR-CONICET. Instituto de Investigaciones en Ciencias Agrarias de Rosario – Universidad Nacional de Rosario – Consejo Nacional de Investigaciones Científicas y Técnicas. Campo Experimental Villarino S2125ZAA, Zavalla, Santa Fe, Argentina.

[2]Cátedra de Genética, Facultad de Ciencias Agrarias, Universidad Nacional de Rosario. Campo Experimental Villarino S2125ZAA, Zavalla, Santa Fe, Argentina.

[3]Plataforma Agrotecnológica Biomolecular - Facultad de Ciencias Agrarias, Universidad Nacional de Rosario. Campo Experimental Villarino S2125ZAA, Zavalla, Santa Fe, Argentina.

[4]Cátedra de Química Orgánica, Facultad de Ciencias Agrarias de Rosario, Universidad Nacional de Rosario. Campo Experimental Villarino S2125ZAA, Zavalla, Santa Fe, Argentina.

*Correspondence: jpereira@unr.edu.ar; Tel.: +54-341-528-8940; Fax: +54-341-528-8940

Figure S1. Amount of *cis*-regulatory elements into promoter regions of differentially expressed in fruit ripening on plant (red circle) and shelf (green circle). The *cis*-regulatory element types shared by both groups are indicated by the number at the intersection of the circles.

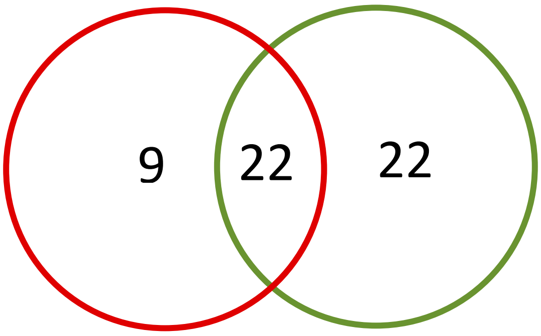

Figure S2. Transvers sections, whole fruits and an image of the vines of the four evaluated genotypes. a- Caimanta cultivar of *Solanum lycopersicum*, b- NOR: accession 804627 (natural mutant in nor gene) of *S. lycopersium*, c- LA1385: accession of *S. lycopersicum* var. *cerasiforme*, d- LA0722: wild accession of *S. pimpinellifolium*

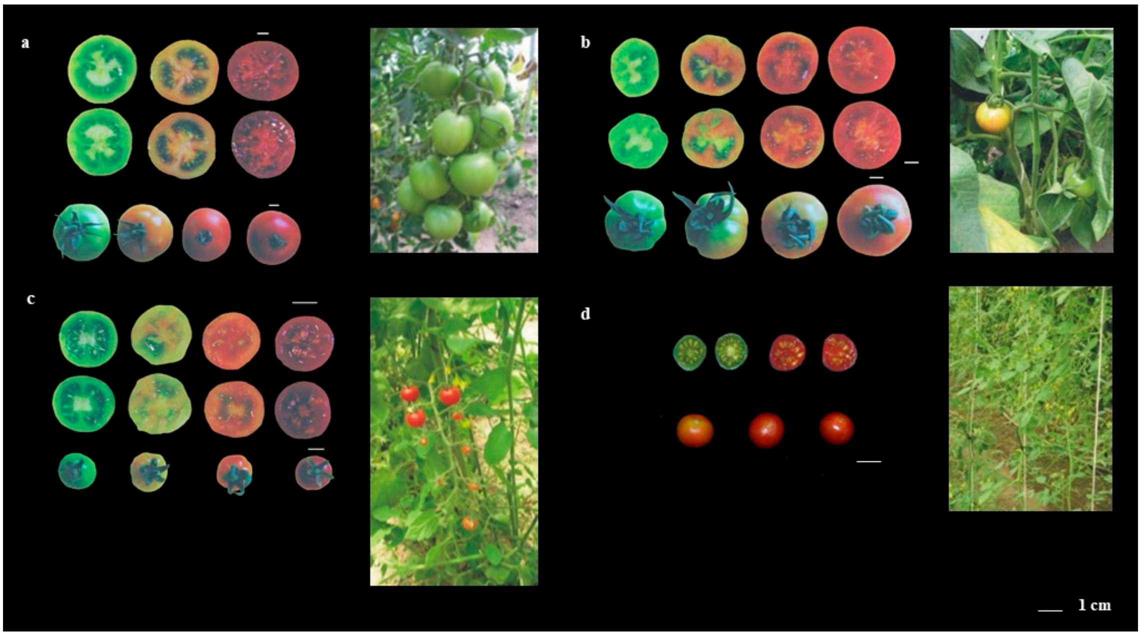

Figure S3. Section of a 5 % denaturing polyaclrilamide gel visualized with a commercial silver staining kit. The gel shows the cDNA profile obtained for the cultivar Caimanta of *Solanum lycopersicum* from primer combination A (Apo11-Mse37). Each band represents transcript-derived fragments (TDFs). MM: molecular marker from 1000 to 200 base pairs. Lane 1, 3 and 5: band profiles detected for three biological replicates from plant-ripened fruits. Lane 2, 4 and 6: band profiles detected for three biological replicates from shelf-ripened fruits. Lane 7: negative control for PCR reaction.

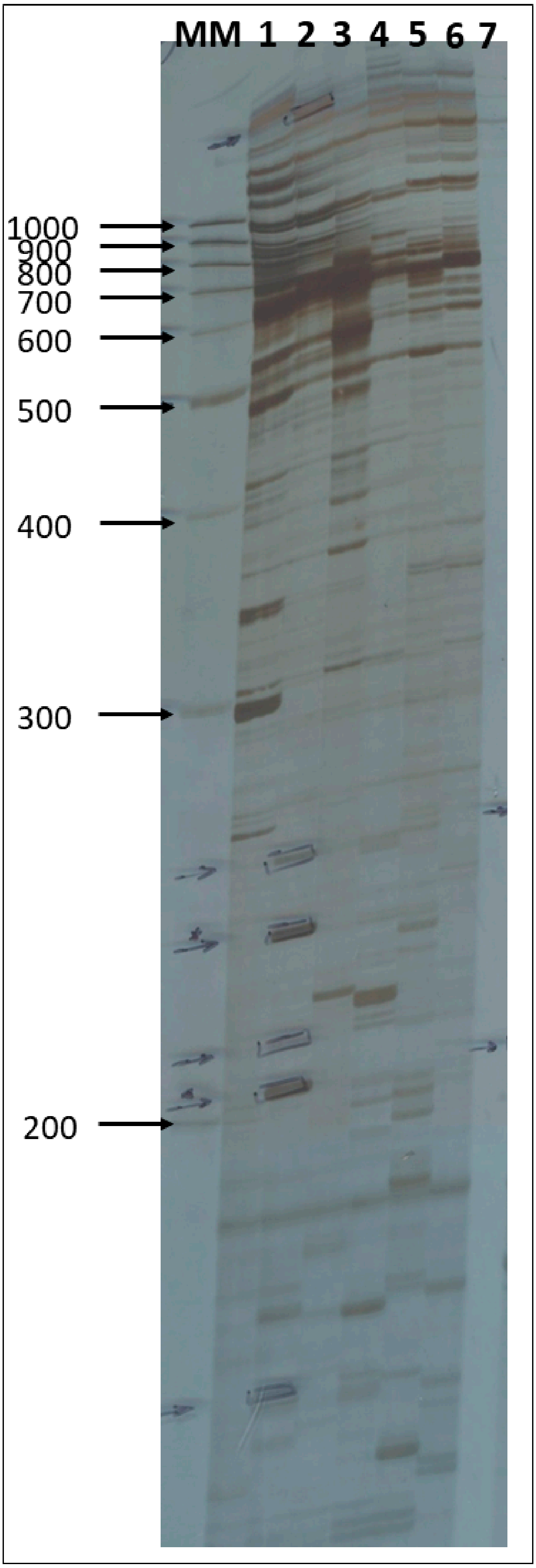

MM 1 2 3 4 5 6 7
1000
900
800
700
600
500
400
300
200

Table S1. Amount of transcript-derived fragments detected by cDNA-AFLP using six specific primer combinations of fruits of four tomato genotypes from plant-ripened and shelf-ripened fruits.

**G.**	Primer Combination A				**G.**	Primer Combination B				**G.**	Primer Combination C			
	% pol	TT	PP	PE		% pol	TT	PP	PE		% pol	TT	PP	PE
**CAI**	43.9	157	25	44	**CAI**	60.0	115	40	29	**CAI**	44.2	120	24	29
**LA0722**	34.7	118	27	14	**LA0722**	37.5	88	17	6	**LA0722**	40.3	139	42	14
**LA1385**	44.6	130	37	21	**LA1385**	41.0	83	25	9	**LA1385**	40.2	117	27	20
**NOR**	47.9	120	37	20	**NOR**	50.4	115	32	26	**NOR**	49.0	145	41	30
**Total**	42.8	525	126	99	**Total**	47.2	401	114	70	**Total**	43.4	521	134	93

**G.**	Primer Combination D				**G.**	Primer Combination E				**G.**	Primer Combination F			
	% pol	TT	PP	PE		% pol	TT	PP	PE		% pol	TT	PP	PE
**CAI**	46.8	47	17	5	**CAI**	41.2	136	34	22	**CAI**	25.2	119	20	10
**LA0722**	59.6	47	17	11	**LA0722**	27.0	252	41	27	**LA0722**	35.6	236	47	37
**LA1385**	38.2	55	6	15	**LA1385**	30.1	186	35	21	**LA1385**	28.3	152	22	21
**NOR**	58.3	115	16	44	**NOR**	30.8	211	35	30	**NOR**	27.8	176	34	14
**Total**	50.7	264	56	75	**Total**	32.3	785	145	100	**Total**	29.2	683	123	82

Primer combination A: Apo11-Mse37. Primer combination B: Apo11-Mse38. Primer combination C: Apo12-Mse37. Primer combination D: Apo12-Mse38. Primer combination E: Apo13-Mse37. Primer combination F: Apo13-Mse38. G.: Genotypes. CAI: cv Caimanta of *Solanum lycopersicum*. LA0722: LA0722 accession of *S. pimpinellifolium*. LA1385: LA1385 accession of *S. lycopersicum* var. *cerasiforme* and NOR: nor mutant (804627) of *S. lycopersicum.* % pol: percentage of polymorphism. TT: total number of transcript-derived fragments (TDFs). PP: exclusive TDFs of plant-ripened fruit. PE: exclusive TDFs of shelf-ripened fruit.
