# Supplementary material for: Genetic Variability of Gene Expression in Tomato Fruits Ripened on and off the Vine: Cis-Regulatory Elements Associated with Differential Transcription Patterns in the Most Discrepant Variety
Source: Plants (Basel). 2025 Dec 24;15(1):53. doi: 10.3390/plants15010053 (PMC12787370; doi:10.3390/plants15010053)
Supplement: Supplementary file 1 [file plants-15-00053-s001.zip › Table S1.pdf]

Genetic variability for gene expression in tomato fruits ripened on and off the vine: cis-regulatory elements are associated with differential transcription patterns in the most discrepant variety

Javier Pereira da Costa[1,2,*]; Eduardo Souza Canada[3]; Ana Ochogavía[1,4]; Gustavo Rodríguez[1,2]; Guillermo Pratta[1,2]

[1]IICAR-UNR-CONICET. Instituto de Investigaciones en Ciencias Agrarias de Rosario – Universidad Nacional de Rosario – Consejo Nacional de Investigaciones Científicas y Técnicas. Campo Experimental Villarino S2125ZAA, Zavalla, Santa Fe, Argentina.

[2]Cátedra de Genética, Facultad de Ciencias Agrarias, Universidad Nacional de Rosario. Campo Experimental Villarino S2125ZAA, Zavalla, Santa Fe, Argentina.

[3]Plataforma Agrotecnológica Biomolecular - Facultad de Ciencias Agrarias, Universidad Nacional de Rosa-rio. Campo Experimental Villarino S2125ZAA, Zavalla, Santa Fe, Argentina.

[4]Cátedra de Química Orgánica, Facultad de Ciencias Agrarias de Rosario, Universidad Nacional de Rosario. Campo Experimental Villarino S2125ZAA, Zavalla, Santa Fe, Argentina.

*Correspondence: jpereira@unr.edu.ar; Tel.: +54-341-528-8940; Fax: +54-341-528-8940

Table S1. Amount of transcript-derived fragments detected by cDNA-AFLP using six specific primer combinations of fruits of four tomato genotypes from plant-ripened and shelf-ripened fruits.

G.	Primer Combination A				G.	Primer Combination B				G.	Primer Combination C			
	% pol	TT	PP	PE		% pol	TT	PP	PE		% pol	TT	PP	PE
**CAI**	43.9	157	25	44	**CAI**	60.0	115	40	29	**CAI**	44.2	120	24	29
**LA0722**	34.7	118	27	14	**LA0722**	37.5	88	17	6	**LA0722**	40.3	139	42	14
**LA1385**	44.6	130	37	21	**LA1385**	41.0	83	25	9	**LA1385**	40.2	117	27	20
**NOR**	47.9	120	37	20	**NOR**	50.4	115	32	26	**NOR**	49.0	145	41	30
**Total**	42.8	525	126	99	**Total**	47.2	401	114	70	**Total**	43.4	521	134	93

G.	Primer Combination D				G.	Primer Combination E				G.	Primer Combination F			
	% pol	TT	PP	PE		% pol	TT	PP	PE		% pol	TT	PP	PE
**CAI**	46.8	47	17	5	**CAI**	41.2	136	34	22	**CAI**	25.2	119	20	10
**LA0722**	59.6	47	17	11	**LA0722**	27.0	252	41	27	**LA0722**	35.6	236	47	37
**LA1385**	38.2	55	6	15	**LA1385**	30.1	186	35	21	**LA1385**	28.3	152	22	21
**NOR**	58.3	115	16	44	**NOR**	30.8	211	35	30	**NOR**	27.8	176	34	14
**Total**	50.7	264	56	75	**Total**	32.3	785	145	100	**Total**	29.2	683	123	82

Primer combination A: Apo11-Mse37. Primer combination B: Apo11-Mse38. Primer combination C: Apo12-Mse37. Primer combination D: Apo12-Mse38. Primer combination E: Apo13-Mse37. Primer combination F: Apo13-Mse38. G.: Genotypes. CAI: cv Caimanta of *Solanum lycopersicum*. LA0722: LA0722 accession of *S. pimpinellifolium*. LA1385: LA1385 accession of *S. lycopersicum* var. *cerasiforme* and NOR: nor mutant (804627) of *S. lycopersicum.* % pol: percentage of polymorphism. TT: total number of transcript-derived fragments (TDFs). PP: exclusive TDFs of plant-ripened fruit. PE: exclusive TDFs of shelf-ripened fruit.
