# Supplementary material for: Genetic Variability of Gene Expression in Tomato Fruits Ripened on and off the Vine: Cis-Regulatory Elements Associated with Differential Transcription Patterns in the Most Discrepant Variety
Source: Plants (Basel). 2025 Dec 24;15(1):53. doi: 10.3390/plants15010053 (PMC12787370; doi:10.3390/plants15010053)
Supplement: Supplementary file 1 [file plants-15-00053-s001.zip › Table S3.pdf]

Genetic variability for gene expression in tomato fruits ripened on and off the vine: cis-regulatory elements are associated with differential transcription patterns in the most discrepant variety

Javier Pereira da Costa[1,2,*]; Eduardo Souza Canada[3]; Ana Ochogavía[1,4]; Gustavo Rodríguez[1,2]; Guillermo Pratta[1,2]

[1]IICAR-UNR-CONICET. Instituto de Investigaciones en Ciencias Agrarias de Rosario – Universidad Nacional de Rosario – Consejo Nacional de Investigaciones Científicas y Técnicas. Campo Experimental Villarino S2125ZAA, Zavalla, Santa Fe, Argentina.

[2]Cátedra de Genética, Facultad de Ciencias Agrarias, Universidad Nacional de Rosario. Campo Experimental Villarino S2125ZAA, Zavalla, Santa Fe, Argentina.

[3]Plataforma Agrotecnológica Biomolecular - Facultad de Ciencias Agrarias, Universidad Nacional de Rosario. Campo Experimental Villarino S2125ZAA, Zavalla, Santa Fe, Argentina.

[4]Cátedra de Química Orgánica, Facultad de Ciencias Agrarias de Rosario, Universidad Nacional de Rosario. Campo Experimental Villarino S2125ZAA, Zavalla, Santa Fe, Argentina.

*Correspondence: jpereira@unr.edu.ar; Tel.: +54-341-528-8940; Fax: +54-341-528-8940

Table S3. Sequences of differentially expressed transcript-derived fragments (TDFs), eluted from polyacrylamide gel and obtained through cDNA-AFLP profiling with six primer-specific combinations, are listed below. Each TDF name was codified as follows: the first three letters indicate the genotype — CAI for the Caimanta cultivar of *Solanum lycopersicum*, CE for the accession LA1385 of *S. lycopersicum* var. *cerasiforme*, and NOR for the cultivar 804627 of *S. lycopersicum* homozygous for the *nor* mutant gene. The abbreviations EST and PL denote whether the TDF is expressed in shelf-ripened fruit or plant-ripened fruit, respectively. The letters A to F refer to the primer combination used, and the numbers indicate the order of elution.

CAI-EST-A-1

ACNNNNNGNCNGATCATATCTGATATTTTATCGTTTGGATATTTTCAACTTTTTCA
CATATCACTCTCATGATTATATACTGTTGAAAATCAAATATATAAAATACTGTTTT
TCTTTTTTTTAAACAGTATGCAAGCATTGAGAAAAACGGTGAATTTTTCATGTCCC
CCAATGACTTTGTCACTCGATACTTGAACATTTTTGGAGAAAGCCAGCCTAATCC
AAAGACTGTGGAACTTTTAAGTGGAGTGGTGGATCAGACCAAAGATGGGTATGTT
TATTTTTAATTATTTTATTTAATTCTTTTTGTTTGAAACAGCTTTCTTTTGCTCTAAT
GACCAGGAAGTCATTAAATGTATACCTTCCCATCTCTTAGGACCAACTCCCACCT
GTTCTTCATTCAATCCATAGAATCGGGGATAACGCCGGAAAGAACATGTGAGCAA
AACGCCAGCTAAAGGTCAGGAACCGTAAAAAGGCCGCGTTGCTGGCGTTTTCCAT
ACGCTCCGTCCCCCTGATGAGCATCACAAAAATCGACGCTCAAGTCAGAGGTGGC
GAAACCCGACAGGACTATAAAGATACCAGGCGCTTCCCCCTGGAAGCTCCCTCGT

GCGCTCTCCCTGTTCCCGACCCTGCCGCTTACCTGGATACCTGTCTCGCCTTTCTC
CCTTTCGGGAAAGCGTGGCTCTTTCTCATAGCTCACGCTGTAGGTATCTCAGTTCG
GTGGTAGGTCGTTCGCTTCCAAGCTGGGGCTGTGTGCACGAAACCTTCCGTTCAG
CCCGATCCGCTGTGCCTTATCAGGGAACTATTGGTCTTGGAGTCCTGCCCCGGGT
AATGACACGTACTTTACGCTACTGTGCAGCAGTCATCTGGGTAATCAGGATTAAT
CAGATCGTGGNATGGTAGGCGGGTGCTACCAGAGATCCTTGAAGGTGTNGGCCT
AACCTACCGGCATCCCAGAAGAACCAGTATTTGGGAATCTGGCTCTCATGCTGAA
GCCAGTTACCATTCGGAAAAAAGGAGTCGGTAGCTTCTTGAATCCCGGCAACCCA
AACCCAGCCCGTCTGGGTAATGCGGTGGTTTTTTTTTCGTGTTTTGTCAAG

CAI-EST-A-2

CNNNNNANNGNNACTTATGGATCTAGACTTTTTATGACCCGAACAAAGAACGAA
GAGTACTGTGGGGATGGATTGGGGAAACTGACAGTGAATCTGCTGACCTGCAGA
AGGGATGGGCATCTGTACAGAGTATTCCAAGGACAGTGCTTTACGACAAGGAGA
CAGGGACACATCTACTTCACTGGCCAGTGGAAGAAATTGAAAGCTTACTCGGGAC
TCCACAAATTGTCCGGGCGGGTCTTTGGAAGGCTGTGGTGTCAATATTTCCCATA
AAAATGCCCAAACCCGATTGGAAGCCAGTTATTTTCTGCCCACTCAAAGAAATGG
AAGAAATCTGCCTCGCTGCGCTCGAAAGTTTATACTGCGGCGAGCGGTATCAGCT
CACTCAAAGGTGGCTATACGGATATCCACTGAATCCGGGGATAACCCAGGAAAG
AACATGTGAGCAAAACGTCAGCTAAAGGTCAGGAACCGTAAAATGGCCGCGTTG
CTGGCTTTTTTCCATCTGCTCCGCCCCCCTGACGAGCATCACAAAAATCGACGCTC
AGTCAGAGGTGGCGAAACCCGACAGGACTATAAAGATATCAGGCGTTTCCCCCT
GGAAAGCTCCCTCGTGCGCTCTCCTGTTCCGACCCTGCCGCTTACCTGGATACCTG
TCCGCCTTTTCTCCCTTCGGGAAGCGTGGCGCTTTTCTCATAGCTCACGCTGTAGG
TATCTCAGTTCGGTGTAGGTCGTTCGCTTCCAGCTGGGCTGTGTGCACGAAACCCC
CCGTTTCAGCCCGAACCGCTGCNCCTTAATCCGGGAACTATTCGTCTTGAGTCCA
ACCCGGTAATGACACTACTTATTCGCTCTCTTGGCAGCAGCTCATCTGGTAATCAG
GATTAT

CAI-EST-A-3

NNNNNNNNTAGCAGATGCATGCGCATCTACGCAGATGCGGAAACTAGATCAGAT
TCCGATTTCTTTCATTTGGGGTTAACACTAATACCGAAACAAATCGAAAGCCATA
CGTTGGCGGCTCGGGGCTCGGGATTCTGATGTTTTCGATGACGGCTCACCGGGA
CGGACCGACGAAAGCCTCCGATTTTGCTTACTCAGGACTCATCAAGTGGGGGTTC
TTTCGTATGCATTCTGTCATGCGATCGGTGGGAGTATGAGTCGAATAATTGAATG
AAAATGGCCTATCCACCCCCGTAAACTGGTTGCGTCAGCCCAAAAAATGAAATGG
GTTAAGTTTGGTTTAATGCAAGAAGAAAAGTTTTGATTTAATGGATGCACTTAAT
CAAATAATTGTGTCTATCCTGCATTTGCTTCACCGCTTGCATCATCTAACTTCCCC
CATTTTCAACCCACCGTTCGCTTACTTTCTTTGCCTCTCATTGGGTACTCTTTCTCT
TTCCTTTCCCTCTCCCACCGTNNGNTTATAAAAACAAACA

CAI-EST-A-4

AANTNNNNCNCNNACGCATCATTACATGAGCAGGTTCCAATCCTATTTGGAAGAC
CACCTATCAACAACAATAACAACTATGCCTCAGTACAAAACAAGTTGAGGGCCA

GTATGCTTTGGATCCTAACTATTACATGTGCAGGTTCCAATCCCACAAATAATGTA
CTCCTTACTCAGGACTCATCAAAATATCGACAATACTTGTTTCCGGTTCCGGTTTG
GCCTTGTTCAACCTGTTTTTCCTTCGGGTGTTGTATGAGTGAGTTGCTAGAATGCC
AACCCACTAACCACCTTCCCGGACTGGATGCTATACCACAAAACTTGAAATGGGA
GAGGTTTGGGTCTATTTGGTAATCGAATATTTGATGGATGGATGCACCTAATCAG
TTAACCGTTTCTATCATGCATTTGCTCGGCCGCTTGCATCATCTCAACTTAGCCAT
GTGCAACTTATTTCTTTTCTCTTTCTTTGCTCTTCATGTGCTTTCTTTCTCTTTTTTT
ATCCTCTCTCTCGGCAGTTTTGAGACACAACACCACCCCCCCCCGCTCAAGTCAG
AG

CAI-EST-A-5

GNAATNAAAGANGGGAGTTGCTTCATCCATACTAAGATTTTGTTCCTCCTCAAAA
GGACCCTTCCTAAACAGAAGAGTTGTCGTTGCCCCATACTCCAGTTTTCACCTTCA
TCACTGACAGAGGAGTCTAATAGGTTTGGAAGTGTACTAATTGTAGCCTTACTCA
GGACTCATCAACTCTTTTTATTGGGCGGCAATACATAGGTCGGGGGTGTTATTG
GGTTTATTCTATCTGTTCTCCCATCGGATGATGTGTGAGAAGATTGTGTTCCAATA
GGGCAACCCAAGACCCGAACACTCCTTTACTACACACATAAAAAAATGTTAGAA
AATTGGTTTTTTTCACAAAGATAATAATACATTTTTAATGACATGCTGTGATCTGT
GTGGCTTTGTTTTATGCATGGGTTCCGCCGCTTGCTTGATCTAACCATACTTATCT
CTCACCCACTTTTACTTCTCTTTATTTC

CAI-EST-A-6

NNNNNNTANATAANTTTGCCATTCATCACTGCTACTGCAGATGGTCCGAAACATA
TTGACACAACATTTACAAGGGCCAAGTTTGAGGAGTTATGCTCAGACTTATTGGA
CAGGCTTACTCAGGACTCATCAAACTTGGAACTTGACTATGTGGTGTCTATCTGTT
GAGGCCTCACTGCAGTAGCAGTGATGAATGGCAAACTGATATTAGTTTGAGTCAA
GGTTGACAATTGGTACGCAGTCAACTACGATGTGTGATTTAATTGCTTGCCTACC
ATCCCACCAACCACCTCCCCTGCCTGTATTCATCACCAAAAAAAATAATTGAAAT
AAAGGTTCCTTCATGCAAATGGTCATACATTTGCTGAAGCAACTAATCACACCAA
TGTACCAATCCTGCTCCTACATTGCATTCATCCCCTAATCTTTTTTTAAACCTAGCC
AGCTAACACTCCCTTCCCTTACCCTTCCCGATCATCAT

CAI-EST-A-7

NNNNNGGANTGGTCGTCNAGCGCTACTCTCAGTTACTTCTCCAGCTAGCATGGAG
ATGATATCAAGAAAATCTGGGGTGTTGCTTACTCAGGACTCATCAACTTCAAGCG
GATTGCTACCGGACGTTCCACCTGCTAATGTGGTATCCCCCCTTGTGGAAATCCTG
ACCCTTAACATGTTGTGGAGGCAAGAGTCCAATTCAGGAAGGTGGTTGTTTGGTA
TTTTTGGGTGTCTCACGCTGACGTCGCACGATAGAGAAACAAGCTAATATGGCCA
AACCGAACCGGAAACATGTATTGTCCACCCAAAAAAATGTTTGAAAGAAATGGA
TTTTAATGAAAAAGGAAATGTATAAGCTGAAGCGATGGATGGCAGTTACCGTTTA
AGACTGGGCGAAAAGGAATTCACCGGTAGTCTAGCTTGCTAACCACTTAGTAATC
CCAACTTGGCTTCATCTCACGTGCATCCGATTGTTAACTATCTCTAGCCATTCGCA
TCACACCGCTGTGGGTAAGGTTC

CAI-EST-B-1

GGNGGTACNGTGTNTTCTATCTCTTGGTACCTTTGAGCACAGAGGCCTTTGCTGTT
CAAGATCACTGGGATATGGATCCAACGGTTACTTGGGAATCATCAGGAGAAGAC
CATGTCTGACCTTTATCCCTCTTCTGTAAGGTTACATGCAGTATATCATCCTCTAG
AGTCCAGAAGGAACAATCGGTCTTCACTGGGTTCATCAGATCATGATTACTCAGG
ACTCATCAACAGTGTGTGTACTGTTGGAACCTTGTTGTTTTGGTTAATAGTCAGAC
TAGGTTCTCTCCAAAAAAGTGTACAGGTGCTTTTGGTCCACCCACTCCTTTTTTTG
TTCCTTTATTTTTACATACAGATCTT

CAI-PL-B-2

CNGGAATGNGNGNNTAGTTTACTGCAGTGTAACGAAGTTGAAGAATAGCTTTTTC
AAACCAATCTTGTCTACAGAAGTATAGTTATGCTCTAAAATCTCTGAGAAATTGC
GAAATCAATTGCTCTGTAACTAAACTTATTTCAATAATTGAAGCAAGATTTTCCAT
TACTCAGGACTCATCAAACAGGGTGGGATGGCACAAAAAAATTCTGATGGCTGTT
TTTTCATCACTAATCTGTGTGTCTTCCCGAACCCGTGCCCGTTTATGCGAGTCTGT
CCTTGGC

CAI-PL-C-1

GNNNTGCTNNNNNGNGGCNTGANTGCGCCGGGCGCTGGAGATCGTGCGGGAGCA
GGCGCCGGATATCGAGGCCGATGGCGAGATGCATGGCGACTGTGCGCTGGATGA
AGGGCTGCGCGCGAAGCTGCTGCCGATGTCGCCGTTGAAGGGGGCGGCGAATTT
GCTGGTTTGTCCGAATGTGGATGCGGGGAATATTGCGTACAACCTGCTTACTCAG
GACTCATCAAATCTGTTCTGAGGCTTTTACCACTCCTGTCTTTTTATTTGCTGGAC
AGCCCCGGCGTCCTATGCCCAAGAAATACTAATGTTTGGCTCTGCAAGATTTTTTT
TTTTTCTTTTTCCATGATAAGATAAGTT

CAI-PL-C-2

CNNGANNNTCNCTGGNTCNGATGGGTTGCGCGCATGTGCGCGACGTAGGTGTCGT
AGTCGGGCAGGCCGACCATCAGCCGCAACGCCTGCCCGAGGTAACGCCCCGCGC
TGCGCAGGTCGCCGCCAAGATCGCTGAACATCGCGGCCTCCCCTTACTCAGGACT
CATCGATGGATTTTTTATTTTTTATCGAATGAGAAAACAAAATCATGATTAGTGTT
TTAATATCACTGTTCGGTGATTCTATATAAACCCCTGCGCTTCTAATACGAGTCTG
ATTCTCA

CAI-PL-E-1

ANNNNNNNNCNTTCCNCAATCTGATCGGGTCCGACAGTCTCTGTCAACATTGTAC
TCTCATCTGTGCTACTCTCAAGATTTGCTATTGCAGATTCTACTTCTTGGATGGCA
CCGTATCTAAGATCTGCTGCTCTAGCAAGATCATATCTCCTTTCAGCTTCTTGTAA
AGCATATGTAAGTTCATCGCGCTTTTGCTTACTCAGGACTCATCAAAATTTTGCGG
TCGCCATTCTGGCTACTCTCCGATAATACGCCGGATTAAAAGACAGTCTGAATAT
GCCCAATCCCGAGAAGTTTACAAATGTTTTTTGCCCAAAAAAAGAAATTTTTGAA
TTTTTGCTTTATGCACTCGGGTGTTTTTTTTTTTGAGAGAGGTATGTCTTCATTTAA
TTGTGCATATAA

CAI-PL-F-1

AANNAANNNNAAAACTAGCTAATCGCGAGAAGAGGACATGTGGATTGAACTCCA
TGGCAAAGGCGCCAAATCTTTCTCGACAATAGCCTTCTCAATCGTCTGTTGAGATT
CCTCATTAAAACCGTCAATTTCGATGAAAAAGAGGTCCTTTGTCAGTGAACTTGC
CATTACTCAGGACTCAACAATCCTCAACCGCACCCTCTTCGTCTTTCTTGGGCTCA
TCATCTTCATCATCACTGATCTCTTTCTCAGTAGTCTTCTCTGTCCAAAGGTAAAT
AGGGTAGCTAATGAATTGGTACGCAGTCAGGGATAACTGACACTTCTTTTTTTTT
CATCATATACCACAATTATATATCTCTCTCGGGCTGCTGGAGAGCAAAAAATTAT
AAAAAATCATTGGGGCGCCAACACAAAATTCAAAAAACACTATTTTATTTGTTTT
TAAAAAAAAAACCTTCCTCCTTCTGCAATATGTTCCACG

CE-EST-C-1

GNNNNGTNNNCCAANNGTAANCGACAGAGGAGTCACCACAGTGTATCGTCAGCA
GCGATATCGCCCCCGAACCATCGAAACAGCGGTTTCAGCGACTGCGTCCGTCGAA
GACATCGTACAACCTGGGCATGTATTCCCGCTTGTGGCACAAAATGGCGGTGTGC
TACACCGTGCCGGTCATACTGTCTCCGGCTGTGACCTAACACGCTTGGCAGGACT
TGAACCTGCCGCAGTGATTTGTGAAATTATCAAATAAGATGGCGAAATGGCGCGC
CGTGATGACCTAGAGATTTTTGCCGAAAAACATGGCTTACTCCTGACTCATCAAA
TCATCAATCTTTGCCTCGCTTAGCTCGTATGTCCGGCTGCGGCGAGCGGTATCAAC
CCACTCAAAGGTGGTTATACGGCTATCCACAGAATCTGGGATAACGCAGGAAAG
AACATGTGAGCTATAGGCCAGCTAAAGGTCAGGAACCGTCAAAAGGCCGCGTTG
CTGGCGTTTTTCTTAGGCTCCGCCCCCCTGACGAGCATCATATAATCGTCGCTCAA
GTCAGAGGTGGCGAAACCCGACAGGACTATAAAGATACCAGGCGTTCCCCCTGG
AAGCTCCCCTCGTGCGCTCTCCCTGTTCCGACCCTGCCGCTTATCGGATACCTGTC
TCGCCTTTTTTCCCTTTGGGGAAAGCGTGGCGCTTTCTCATAGCTCACGCTGNACG
TATCTTCAGTTCTG

CE-PL-A-1

GGNNNTNGNNNANNNATCGTGTTCGGCACCGATTCGCCGCCGAACGAGCCGGGT
ATGTGGCTGCGCGAACTCGAAATGTTGTGCGGGCCGGCACCGCAAGGCATGGAT
CTGGATGAGGACGGTCTCGAAGACTATATGGGCAACAACATCGCCCGTCTGGTCG
GTATCGAGCCGACCAAGCCGCCGAAGGATCTGGCCGAAGCGGAGAAGCGTTTGA
AGGCGACTTACGTGAACGACGTAACGCAGCCCGCTTACTCAGGACTCATCAAGA
ACGCCGCCCCACCGACCCCCTCGCTGTTCTGTAGTTCACCAATAAAAAAATTGGT
TGCTTGTGTGTTTTCGTGAAGATGGTAATCTATCTTTTGAGGGAATAGATGCTCCT
TATCTATTAACCCTGTCTATCATGCATTTGTGCGGTCGCTTGCCTTATCCCTGCTTG
CCCATCTTCACTTTCTTTTCCTTTCTCGTGCTTTGC

CE-PL-A-2

NNGGNNNANNTACGTCTGATGCCGATCTGGAGTGCGAAGACATTACTTCTGATAA
GAAGTGCGACATAGACATGTGGAGTAATGTCGTTTCCTTTGCCACTTTAAAAAGA
CGACGCGAGGATGTCCGTTGCTTTACCCCCCCCACTACCCCTTTTCATCTGCAACA
ACCAATCTCTGCACGAGGTAACCAGCTACTTACTCACGACTCATCAAATCAGTGA

CTGTGGAACTTTTAAGTGGAGTGGTGGATCATACCAAAGATGGGTATGTTTATTT
TTAATTATTTTATTTAATTCTTTTTGTTTGAAACAGCTTTCTTTTGCTCTAATGACC
AGGAAGTCATTAAATGTATACCTTCCCATCTCTTAGGACCAA

NOR-PL-A-1

NNGCGTNCTGNTCNATAACAGGACTCCGAGGAAGAGGACATCTCCACCACATGG
CTCGCCCCTCAAGAAGGGCAACCTGGAAAAGGAACCAGACTCTGTCTCTCCGTCG
TTACCGTTGATCTGCTTGACCTTTTTGAGAGGAGTGTCGCTTGCTTACTCAGGACT
CATCACAAGCTCTTCAAGTCGTCTCCACTAGTTGCGGGTTCTTGATTGGCCATTTC
CTCTCTGATTTCCCTTTAGCTGGGGGTGGAGAAAGTTTGTTTATTGCCCCCCCACT
AACCACTTCTCTGTCTTTTTAACCAGATAAAAAAAGTTTTTGAACTTGTGGTTTTT
CATAAAAATGTTAATTTATTATTTGATGGATGAATGCACCTTATGTCCTAACCTTT
TCTATCATGTATTTTTTTCGGTCGCTTGCGTCATCCCAACTTACTCAGGTCCCTACT
TCCTTTCACTTCTCTTTCATTGCT

NOR-EST-F-1

ANNNANTNNTNNTTACCGTGTATTATCGGGCCTCTTGACGGGCTCGGCAGTCACG
GCGGGCCTTACGTTTCGACAGAAATGGATCCGGCAGGCCGCGCTCGATTGTGCCC
GAGCAGCTTGATGGATGTGACTTGAGCGCATTACTCAGGACTCATCCACATTAGT
AACGTAGCTAACGCGTGAAGTTGACCGCCTGAGGAGTACGGTCGCAAGATTACTC
AGGACTCATCAAGAAAAGAGAGTTTTTTTAGGAAAACTTTTTTTTTTCTCCAAA
CACCCCCCCCCGCGGAAAAACACAATAAAAAAACTAAAGGGGATTACCAACACT
GTTTTGTTTCTCCCCTATATAAAACCCCTTATAGTTCTCCTATCCCTTTCTGATGAG
ATAATATCTATAAAAAAGTAAGGGGGCCCCACCCTTTTTTTAAAAACATCCTTTT
A

NOR-PL-E-1

ANAANNNNNNNAATTTCGGAGGACGCTCGAGACGCCGGTGCAAGCGGCTCGTCG
TCCGCCAGCTGGCGTGCGCACACGGTCAATTACAAATGGCTTTCCTTACTCAGGA
CTCATCAAAGTCTTGTGTGATGGCGCCTGCTTTTGCTGGGGGCCTGCCTTCGTGGA
GAGATCTACTATGTCCTTAATGAGCTGACTCAGGACTCATCAAATGGTTGTTTTTT
TATCCCTTGCCGGATGTTGATCCGAAATACTTGGCGTTAAAGAGAGTGTGGAGAC
CCAAACTCAAAAAACCAAAAAAATATTTTTAGACCACAAAAAAAAAGTTAGAAC
TTTTACTTAATCGCTCGATGTTTTTTGCTTTGCTGACTAATAAGTCATTTTTATGAG
CTG

PIM-PL-E-1

ANNNGNCNANGGAACNACCTGGAGTCGGCCGCCATATCTATATACCTGATCTGG
GTGGTGGTGCTCGGTGCGGTGATTTCTGTTCAAAACCTGCTTACTCAGGACTCATC
AGTTGGCGGCTCCTGGCGCGTGATTCTGATATTTTTCCATGACACGTCGCCGGGA
CGGCCCGCCGAAAGCCTCCGATTTTGCTTACTCAGGACTCATCCAATCATCAAGT
TGACCTCTCTATTCTGTTTGCCCGGGCTGAAAATGGCGGGTCAAAAGAGACAAGT
AATGTCCCGAGGCGTCCATGTTGTAGTTTTGTTTTTTGGGTGTTAAAAATTTTGGT

TGTTCATTGCCCTTCTGCGGTGGGGTGTTTTTGGCTTTGGGGGGAATTTGTGTTTTT
AATTGTGGGT
