# Supplementary material for: Genetic Variability of Gene Expression in Tomato Fruits Ripened on and off the Vine: Cis-Regulatory Elements Associated with Differential Transcription Patterns in the Most Discrepant Variety
Source: Plants (Basel). 2025 Dec 24;15(1):53. doi: 10.3390/plants15010053 (PMC12787370; doi:10.3390/plants15010053)
Supplement: Supplementary file 1 [file plants-15-00053-s001.zip › Table S4.pdf]

Genetic variability for gene expression in tomato fruits ripened on and off the vine: cis-regulatory elements are associated with differential transcription patterns in the most discrepant variety

Javier Pereira da Costa[1,2,*]; Eduardo Souza Canada[3]; Ana Ochogavía[1,4]; Gustavo Rodríguez[1,2]; Guillermo Pratta[1,2]

[1]IICAR-UNR-CONICET. Instituto de Investigaciones en Ciencias Agrarias de Rosario – Universidad Nacional de Rosario – Consejo Nacional de Investigaciones Científicas y Técnicas. Campo Experimental Villarino S2125ZAA, Zavalla, Santa Fe, Argentina.

[2]Cátedra de Genética, Facultad de Ciencias Agrarias, Universidad Nacional de Rosario. Campo Experimental Villarino S2125ZAA, Zavalla, Santa Fe, Argentina.

[3]Plataforma Agrotecnológica Biomolecular - Facultad de Ciencias Agrarias, Universidad Nacional de Rosario. Campo Experimental Villarino S2125ZAA, Zavalla, Santa Fe, Argentina.

[4]Cátedra de Química Orgánica, Facultad de Ciencias Agrarias de Rosario, Universidad Nacional de Rosario. Campo Experimental Villarino S2125ZAA, Zavalla, Santa Fe, Argentina.

*Correspondence: jpereira@unr.edu.ar; Tel.: +54-341-528-8940; Fax: +54-341-528-8940

Table S4. Relative gene expression tested by RT-qPCR for each gene identified by sequencing of bands eluted from cDNA-AFLP profiles.

**Identifier / Gene**	**ER in PL**	**S.E.**	**ER in EST**	**E.E.**	***p-value***
**Solyc03g083910.2**	2,07	0,37	5,13	1,48	0,0644
Solyc12g044820.1	3,26	1,40	8,28	2,67	0,1272
**Solyc11g020040.1**	4,07	0,71	13,89	4,34	0,0830
**Solyc08g080940.2**	1,51	0,17	2,67	0,22	**0,0013**
Solyc06g076940.2	-	-	-	-	-
Solyc11g020040.2	-	-	-	-	-
**Solyc03g115230.2 / Solyc06g082560.1**	11,72	2,05	1,51	0,20	**0,0006**
Solyc03g112910.2	1,75	0,28	3,42	0,50	**0,0160**
Solyc06g064630.2	1,23	0,06	1,45	0,16	0,2381

ER: relative expression. S.E: standard error, PL: plant-ripened fruit. EST: shelf-ripened fruit. In bold, the significant *p-value* as the genes selected for subsequent analyses. Expression relative to the reference gene SAND.
