# Supplementary material for: Genetic Variability of Gene Expression in Tomato Fruits Ripened on and off the Vine: Cis-Regulatory Elements Associated with Differential Transcription Patterns in the Most Discrepant Variety
Source: Plants (Basel). 2025 Dec 24;15(1):53. doi: 10.3390/plants15010053 (PMC12787370; doi:10.3390/plants15010053)
Supplement: Supplementary file 1 [file plants-15-00053-s001.zip › Table S5.pdf]

Genetic variability for gene expression in tomato fruits ripened on and off the vine: cis-regulatory elements are associated with differential transcription patterns in the most discrepant variety

Javier Pereira da Costa[1,2,*]; Eduardo Souza Canada[3]; Ana Ochogavía[1,4]; Gustavo Rodríguez[1,2]; Guillermo Pratta[1,2]

[1]IICAR-UNR-CONICET. Instituto de Investigaciones en Ciencias Agrarias de Rosario – Universidad Nacional de Rosario – Consejo Nacional de Investigaciones Científicas y Técnicas. Campo Experimental Villarino S2125ZAA, Zavalla, Santa Fe, Argentina.

[2]Cátedra de Genética, Facultad de Ciencias Agrarias, Universidad Nacional de Rosario. Campo Experimental Villarino S2125ZAA, Zavalla, Santa Fe, Argentina.

[3]Plataforma Agrotecnológica Biomolecular - Facultad de Ciencias Agrarias, Universidad Nacional de Rosario. Campo Experimental Villarino S2125ZAA, Zavalla, Santa Fe, Argentina.

[4]Cátedra de Química Orgánica, Facultad de Ciencias Agrarias de Rosario, Universidad Nacional de Rosario. Campo Experimental Villarino S2125ZAA, Zavalla, Santa Fe, Argentina.

*Correspondence: jpereira@unr.edu.ar; Tel.: +54-341-528-8940; Fax: +54-341-528-8940

Table S5. Sequence and function of *cis*-regulatory elements identified in the 1.5 kb upstream promoter region from five tomato (*Solanum lycopersicum*) genes that were differentially expressed in shelf-ripened fruit (1- Solyc03g083910.2, 2- Solyc11g020040.1, 3- Solyc08g080940.2) and in plant ripened fruit (4- Solyc03g115230.2 and 5- Solyc06g082560.1).

Cis-regulatory elements	Organism	Sequence	Function	Shelf			Plant	
				1	2	3	4	5
A-box	*Petroselinum crispum*	CCGTCC	cis-acting regulatory element	-	-	575; +	-	-
AAAC-motif	*Spinacia oleracea*	CAATCAAAACCT	light responsive element	-	-	1239; +	-	-
AAGAA-motif	*Avena sativa*	GAAAGAA<br>gGTAAAGAAA<br>GAAAGAA	Unknown	1060; -	243; -<br>455; +<br>697; +	-	245; +<br>585; -	446; +<br>622; -<br>1437; -
ABRE	*Arabidopsis thaliana*<br>*Triticum aestivum*<br>*Hordeum vulgare*	ACGTG<br>CACGTG<br>GACACGTGGC<br>GCAACGTGTC	cis-acting element involved in the abscisic acid responsiveness	-	-	1056; +<br>1410; +<br>1386; -<br>1407; -<br>1384; +<br>1387; +	698; +<br>699; +	-
ABRE3a	*Zea mays*	TACGTG	Regulatory elements related to abiotic stress response, involved in the abscisic acid response,	-	-	1055; +	-	-
ABRE4	*Zea mays*	CACGTA	Regulatory elements related to abiotic stress response, involved in the abscisic acid response	-		1055; -	-	-
ACA-motif	*Pisum sativum*	AATTACAGCCATT	part of gapA in (gapA-CMA1) involved with light responsiveness	-	-	-	-	361; -
ACE	*Petroselinum crispum*	GACACGTATG	cis-acting element involved in light responsiveness	-	-	-	-	423; +
ACTCATCCT sequence	*Arabidopsis thaliana*	ACTCATCCT	drought response	-	-	1274; +	-	-

AP-1	*Arabidopsis thaliana*	TGAGTTAG	Unknown	-	-	417; -	-	-
ARE	*Zea mays*	AAACCA	Antioxidant Responsive Element; a cis-acting regulatory element essential for anaerobic induction	98; -	365; +<br>1470; +	939; +<br>1078; +	1248; -<br>1441; -<br>1381; -<br>1294; -<br>1433; -	78; +<br>378; +<br>119; +
AT-rich element	*Glycine max*	ATAGAAATCAA	binding site of AT-rich DNA binding protein (ATBP-1)	746; +	-	585; -	-	-
ATC-motif	*Spinacia oleracea*	AGTAATCT	part of a conserved DNA module involved in light responsiveness	-	-	-	-	896; -
ATCT-motif	*Pisum sativum*	AATCTAATCC	part of a conserved DNA module involved in light responsiveness	1092; +	360; +	-	-	-
AT~TATA-box	*Arabidopsis thaliana*	TATATA<br>TATATAAA	core promoter / enhancer element	436; +<br>1451; -<br>951; -	160; +<br>1311; -<br>965; -<br>1389; -	593; +<br>595; +	191; -<br>279; +<br>199; +	706; +<br>969; -<br>266; +
Box 4	*Petroselinum crispum*	ATTAAT	part of a conserved DNA module involved in light responsiveness	582; +<br>826; -<br>660; +	309; +	-	213; +<br>452; +	455; +<br>980; -<br>634; +
CAAT-box	*Pisum sativum;*<br>*Arabidopsis thaliana*<br>*Nicotiana glutinosa*	CAAT<br>CAAAT<br>CCAAT	common cis-acting element in promoter and enhancer regions	1290; +	1058; +	1311; -	683; +	1117; -
CARE	*Oryza sativa*	CAACTCAC	GA response	-	-	506; -	-	-
CAT-box	*Arabidopsis thaliana*	GCCACT	cis-acting regulatory element related to meristem expression	-	-	-	39; -	-
CCGTCC-box	*Petroselinum hortense*	CCGTCC	Cis-acting regulatory element related to meristem specific activation	-	-	575; +	-	-
CGTCA-motif	*Hordeum vulgare*	CGTCA	cis-acting regulatory element involved in the MeJA-responsiveness	-	315; +<br>738; +<br>431; -	395; -<br>398; +	-	-
ERE	*Nicotiana glutinosa*	ATTTCATA<br>ATTTTAAA	Ethylene-responsive element	-	-	1010; +	564; -<br>1338; +<br>1187; -	231; +<br>310; -<br>294; +<br>1166; +<br>233; -<br>771; -<br>308; +
G-box	*Arabidopsis thaliana*<br>*Zea mays*<br>*Lycopersicon esculentum*	TACGTG<br>CACGAC<br>tgACACGTGGCA<br>GCCACGTGGA<br>CACGTC<br>CACGTT	cis-acting regulatory element involved in light responsiveness	-	400; -<br>933; +	1055; +<br>1383; -<br>1407; +<br>1406; -	696; +	-
GA-motif	*Arabidopsis thaliana*	ATAGATAA	part of a light responsive element	-	1291; -	-	-	-
GATA-motif	*Arabidopsis thaliana*	AAGATAAGATT<br>GATAGGA	part of a light responsive element	476; -		-	-	244; -
GCN4_motif	*Oryza sativa*	TGAGTCA	cis-regulatory element involved in endosperm expression	-		-	1; -	-
GT1-motif	*Avena sativa*<br>*Arabidopsis thaliana*	GGTTAAT	light responsive element	1327; -	-	-	-	-
GTGGC-motif	*Spinacia oleracea*	GATTCTGTGGC	part of a light responsive element	-	-	-	-	-
Gap-box	*Arabidopsis thaliana*	CAAATGAA(A/G)A	part of a light responsive element	964; -	-	-	490; +	-
I-box	*Gossypium hirsutum*<br>*Triticum aestivum*	AAGATAAGGCT<br>AGATAAGG	part of a light responsive element	476; -	-	-	-	-
LTR	*Hordeum vulgare*	CCGAAA	cis-acting element involved in low-temperature responsiveness	-	-	-	868; +<br>1398; -	-
MRE	*Petroselinum crispum*	AACCTAA	MYB binding site involved in light responsiveness	-	-	704; +	-	-
MYB	*Arabidopsis thaliana*	CAACCA<br>TAACCA	Cis-acting regulatory element involved in regulation of drought inducible gene expression	304; +<br>1329; +	35; +<br>510; +<br>225; +<br>1268; +	-	23; -<br>182; +<br>70; -<br>1207; -	476; -<br>28; +
MYC	*Arabidopsis thaliana*	CATTTG<br>CAATTG<br>CATGTG	Cis-acting regulatory element involved in early response to drought and abscisic acid induction	968; +	395; -	776; -<br>1020; +	490; -<br>914; +	1430; +<br>71; +
Myb	*Arabidopsis thaliana*	TAACTG<br>CAACTG	Cis-acting regulatory element involved in regulation of drought inducible gene expression	94; +	189;-	523; +<br>559; +	-	189; -
Myb-binding	*Nicotiana tabacum*	CAACAG	cell cycle and cell proliferation	-	-	-	-	476; -

site			response<br>MYB binding site involved in drought-inducibility					
Myc	*Arabidopsis thaliana*	TCTCTTA	Unknown	-	-	-	1136; +	1442; +
P-box	*Oryza sativa*	CCTTTTG	gibberellin-responsive element	1396; +	-		542; +<br>940; -	498; -
RY-element	*Helianthus annuus*	CATGCATG	cis-acting regulatory element involved in seed-specific regulation	-	-	1251; -	-	-
STRE	*Arabidopsis thaliana*	AGGGG	Stress response element. Heat shock protein-related element STRE	-	1170; +	-	1112; +	-
TATA	*Arabidopsis thaliana*	TATAAAAT	core promoter / enhancer element	72; +	-	727; +<br>997; -	-	678; +<br>787; -<br>395; +<br>674; -
TATA-box	*Brassica napus*<br>*Brassica oleracea*	TATA<br>ATTATA<br>ATATAA<br>ATATAT	core promoter element around -30 of transcription start	1385; +	1093; +	1349; +	TATA-less	1241; +
TATC-box	*Oryza sativa*	TATCCCA	cis-acting element involved in gibberellin-responsiveness	-	-	-	649; +<br>924; +	-
TCA-element	*Nicotiana tabacum*	CCATCTTTTT	cis-acting element involved in salicylic acid responsiveness	-	1016; -	-	-	-
TCCC-motif	*Spinacia oleracea*	TCTCCCT	part of a light responsive element	-	-	-	-	1039; -<br>1269; -
TC-rich repeats	*Nicotiana tabacum*	GTTTTCTTAC<br>ATTCTCTAAC	cis-acting element involved in defense and stress responsiveness	1059; +	652; +<br>1091; -<br>1025; -		-	-
TCT-motif	*Arabidopsis thaliana*	TCTTAC	part of a light responsive element	-	1025; -	-	130; -	-
TGACG-motif	*Hordeum vulgare*	TGACG	cis-acting regulatory element involved in the MeJA-responsiveness	-	315; -<br>738; -<br>431; +	395; +<br>398; -	-	
Unnamed__1	*Zea mays*<br>*Petunia sp.*<br>*Glycine max*	CGTGG<br>GCCACGTGGC<br>GAATTTAATTAA	Unknown<br>60K protein binding site	-	438; -<br>939; -<br>932; -	1384; -<br>1408; -	697; -	452; +
Unnamed__6	*Zea mays*	taTAAATATct	Unknown	104; +	-	809; +<br>1140; -	-	276; +
W box	*Arabidopsis thaliana*	TTGACC	Cis-acting regulatory element involved in direct fungal elicitor stimulated transcription of defense genes and activation of genes involved in response to wounding WRKY binding site	-	-	1468; +	106; -	
WUN-motif	*Nicotiana glutinosa*	AAATTACT<br>TTATTACAT<br>AAATTTCTT<br>TAATTACTC	Wound-responsive element	-	469; +	-	-	
chs-CMA1a	*Daucus carota*	TTACTTAA	part of a light responsive element	-	-	1154; +	-	
chs-CMA2a	*Petroselinum crispum*	TCACTTGA	part of a light responsive element	989; +	-	-	-	
circadian	*Lycopersicon esculentum*	CAAAGATATC	cis-acting regulatory element involved in circadian control	-	347; +	-	-	
