# Supplementary material for: Genetic Variability of Gene Expression in Tomato Fruits Ripened on and off the Vine: Cis-Regulatory Elements Associated with Differential Transcription Patterns in the Most Discrepant Variety
Source: Plants (Basel). 2025 Dec 24;15(1):53. doi: 10.3390/plants15010053 (PMC12787370; doi:10.3390/plants15010053)
Supplement: Supplementary file 1 [file plants-15-00053-s001.zip › Table S6.pdf]

Genetic variability for gene expression in tomato fruits ripened on and off the vine: cis-regulatory elements are associated with differential transcription patterns in the most discrepant variety

Javier Pereira da Costa[1,2,*]; Eduardo Souza Canada[3]; Ana Ochogavía[1,4]; Gustavo Rodríguez[1,2]; Guillermo Pratta[1,2]

[1]IICAR-UNR-CONICET. Instituto de Investigaciones en Ciencias Agrarias de Rosario – Universidad Nacional de Rosario – Consejo Nacional de Investigaciones Científicas y Técnicas. Campo Experimental Villarino S2125ZAA, Zavalla, Santa Fe, Argentina.

[2]Cátedra de Genética, Facultad de Ciencias Agrarias, Universidad Nacional de Rosario. Campo Experimental Villarino S2125ZAA, Zavalla, Santa Fe, Argentina.

[3]Plataforma Agrotecnológica Biomolecular - Facultad de Ciencias Agrarias, Universidad Nacional de Rosario. Campo Experimental Villarino S2125ZAA, Zavalla, Santa Fe, Argentina.

[4]Cátedra de Química Orgánica, Facultad de Ciencias Agrarias de Rosario, Universidad Nacional de Rosario. Campo Experimental Villarino S2125ZAA, Zavalla, Santa Fe, Argentina.

*Correspondence: jpereira@unr.edu.ar; Tel.: +54-341-528-8940; Fax: +54-341-528-8940

Table S6. Functional categories to which the *cis*-regulatory elements found in genes with differential expression in tomato fruits ripened on the plant and shelf.

**Functional categories**	**Motifs function**	**Motif name**
**Core promoter / Enhancer element**		AT~TATA-box, CAAT-box, TATA, TATA-box
**Plant development**	**Meristem specific activation**	CAT-box, CCGTCC-box
	**Endosperm expression**	GCN4_motif
	**Responding to cell cycle and proliferating**	circadian
	**Seed-specific regulation**	RY-element
**Hormone regulation**	**Gibberellin-responsive element**	CARE, P-box, TATC-box
	**Involved in the MeJA response**	CGTCA-motif, TGACG-motif
	**Involved in the response to salicylic acid**	TCA-element
	**Ethylene responsive element**	ERE
	**Involved in abscisic acid sensitivity**	ABRE, ABRE3a; ABRE4

**Stress response**	**Light responsive element**	AAAC-motif, ACA-motif, ACE, AT-rich element, ATC-motif, ATCT-motif, Box 4, G-box, GA-motif, GATA-motif, GT1-motif, Gap-box, I-box, MRE, TCCC-motif, TCT-motif, chs-CMA1a, chs-CMA2a
	**Drought response**	ACTCATCCT sequence, MYB, MYC, Myb, Myb-binding site
	**Low Temperature**	LTR
	**High heat**	STRE
	**Injury and defence response**	TC-rich repeats, W box, WUN-motif
	**Anaerobic response**	ARE
**Unknown**		AAGAA-motif, AP-1, Myc, Unnamed__1, Unnamed__6
