# Supplementary material for: Genetic Variability of Gene Expression in Tomato Fruits Ripened on and off the Vine: Cis-Regulatory Elements Associated with Differential Transcription Patterns in the Most Discrepant Variety
Source: Plants (Basel). 2025 Dec 24;15(1):53. doi: 10.3390/plants15010053 (PMC12787370; doi:10.3390/plants15010053)
Supplement: Supplementary file 1 [file plants-15-00053-s001.zip › Table S7.pdf]

Genetic variability for gene expression in tomato fruits ripened on and off the vine: cis-regulatory elements are associated with differential transcription patterns in the most discrepant variety

Javier Pereira da Costa[1,2,*]; Eduardo Souza Canada[3]; Ana Ochogavía[1,4]; Gustavo Rodríguez[1,2]; Guillermo Pratta[1,2]

[1]IICAR-UNR-CONICET. Instituto de Investigaciones en Ciencias Agrarias de Rosario – Universidad Nacional de Rosario – Consejo Nacional de Investigaciones Científicas y Técnicas. Campo Experimental Villarino S2125ZAA, Zavalla, Santa Fe, Argentina.

[2]Cátedra de Genética, Facultad de Ciencias Agrarias, Universidad Nacional de Rosario. Campo Experimental Villarino S2125ZAA, Zavalla, Santa Fe, Argentina.

[3]Plataforma Agrotecnológica Biomolecular - Facultad de Ciencias Agrarias, Universidad Nacional de Rosario. Campo Experimental Villarino S2125ZAA, Zavalla, Santa Fe, Argentina.

[4]Cátedra de Química Orgánica, Facultad de Ciencias Agrarias de Rosario, Universidad Nacional de Rosario. Campo Experimental Villarino S2125ZAA, Zavalla, Santa Fe, Argentina.

*Correspondence: jpereira@unr.edu.ar; Tel.: +54-341-528-8940; Fax: +54-341-528-8940

Table S7. Restriction enzymes, adapters, pre-amplification and specific primer combinations used for cDNA-AFLP profiling of RNA

Restriction enzymes		***ApoI***		***MseI***	
Adapters	**Top Strand**	CTCGTAGACTGCGTACC		GACGATGAGTCCTGAG	
	**Bottom Strand**	AATTGGTACGCAGTCTAC		TACTCAGGACTCAT	
Pre-amplification primers (0)		CTCGTAGACTGCGTACCAATT		GACGATGAGTCCTGAGTAA	
Selective amplification primers (+1)		GACTGCGTACCAATTG (Apo11)<br>GACTGCGTACCAATTA (Apo12)<br>GACTGCGTACCAATTC (Apo13)		GATGAGTCCTGAGTAAG (Mse37)<br>GATGAGTCCTGAGTAAT (Mse38)	
**Combination A**	Apo11-Mse37	**Combination C**	Apo13-Mse37	**Combination E**	Apo12-Mse38
**Combination B**	Apo12-Mse37	**Combination D**	Apo11-Mse38	**Combination F**	Apo13-Mse38
